# Genome-Wide Analysis of the Molecular Functions of B3 Superfamily in Oil Biosynthesis in Olive (*Olea europaea* L.)

**DOI:** 10.1155/2023/6051511

**Published:** 2023-02-14

**Authors:** Jipeng Qu, Bixia Wang, Zhou Xu, Shiling Feng, Zhaoguo Tong, Tao Chen, Lijun Zhou, Zhengsong Peng, Chunbang Ding

**Affiliations:** ^1^College of Life Sciences, Sichuan Agricultural University, Yaan 625014, China; ^2^Panxi Crops Research and Utilization Key Laboratory of Sichuan Province, Xichang University, Xichang 615013, China; ^3^College of Environmental Science and Engineering, China West Normal University, Nanchong 637009, China; ^4^College of Agricultural Science, Xichang University, Xichang 615013, China

## Abstract

The plant B3 gene superfamily contains a large number of transcription factors playing a vital role in both vegetative growth and reproductive development in plants. Although several B3 genes have been well studied, molecular functions of the B3 genes in olive are largely unknown. In our study, a total of 200 B3 genes were identified in olive genome based on RNA-seq and comparative genomic analyses and further classified into five groups (i.e., REM, RAV, LAV, HSI, and ARF) based on phylogenetic analysis. Results of gene structure and motif composition analyses revealed diversified functions among these five groups of B3 genes. Results of genomic duplication and syntenic analyses indicated the gene expansion in the B3 genes. Results of gene expression based on both transcriptomics and relative expression revealed the tissue-biased expression patterns in B3 genes. The results of the comparative expression analysis of B3 genes between two olive cultivars with high and low oil contents identified several potential REM genes which may be involved in oil biosynthesis in olive. Based on the comprehensive characterization of the molecular structures and functions of B3 genes in olive genome, our study provided novel insights into the potential roles of B3 transcription factors in oil biosynthesis in olive and lays the groundwork for the functional explorations into this research field.

## 1. Introduction

As one of the plant-specific superfamilies, the B3 transcription factors (TFs) play critical regulatory functions in plant development, containing at least one B3 DNA-binding domain [1]. The B3 domain performing sequence-specific DNA-binding activities was first identified in corn *VP1* [2] and *Arabidopsis ABI3* [3]. To date, the B3 genes are widely identified in crop and model plants, such as *Arabidopsis thaliana*, *Glycine max*, *Oryza sativa*, and *Zea mays* [4], and classified into five families, i.e., LAV, HSI, RAV, ARF, and REM [5, 6] based on the varied domain compositions [6]. For example, the REM family members contain two B3 domains, whereas the LAV family members have only one B3 domain. Besides the B3 domain, several other typical domains are also identified in the B3 superfamily, e.g., the APETALA2 (AP2) domain in the HSI family and both the ARF domain and auxin/indole-3-acetic acid (Aux/IAA) domain in the ARF family as well as the zinc finger Cys- and Trp-containing domain (zf-CW) detected in the RAV family. The molecular mechanism underlying the regulatory functions of these domains in plant development remains unclear.

In plants, the B3 genes play fundamental roles in multiple biological processes such as regulating plant development and defending against stress responses [1, 7–10]. For example, the B3 genes *ABI3*, *FUSCA3* (*FUS3*), and *Leafy Cotyledon 2* (*LEC2*) in *A. thaliana* LAV family are involved in the regulation of seed development and storage protein accumulation [11–13]. These TFs recognize and regulate seed-specific genes by binding to the Sph/RY motif (CATGCA) in the promoter [14–16]. Furthermore, the HSI family members are known to restrain seed maturation genes ectopically expressed in the sugar signal pathway during seedling development [17]. Recently, a study showed that HSI2 interacted with MSI1 (i.e., one of the components of polycomb repressive complex 2) to regulate seed maturation by repressing AGL15, which was involved in seed development [18]. Moreover, the RAV family members play important roles in floral organ development and stress response [19–23]. In *Arabidopsis*, two members of the RAV family (i.e., *TEM1* and *TEM2*) repress the *FLOWERING LOCUS T* (*FT*) to delay the flowering stage [19]. To date, plenty of molecular and genetic evidence derived from *Arabidopsis* and other plants suggests that the ARF family members are involved in various auxin-mediated physiological processes, such as apical dominance, lateral root formation, vascular differentiation, embryo patterning, and shoot elongation [24–29]. For instance, mutant *arf2* in the ARF family caused delayed phenotypic development, such as flower development and silique development [30]. Although a large number of members are identified in the REM family among plants, few genes have been functionally characterized [31–34]. The first REM gene *BoREM1* isolated from the reproductive meristems in cauliflower was involved in the establishment of the floral meristem [35], while the *Arabidopsis AtREM1* (i.e., a *BoREM1* ortholog) expressed in the reproductive meristem was involved in floral organ development [31].

Olive (*Olea europaea* L.) is a popular agricultural and industrial crop widely cultivated in the Mediterranean region. As one of the major edible oils in the world, the olive oil contains high contents of fatty acids and important secondary metabolites. In the immature olive fruits, the phenolic compound of secoiridoid, i.e., oleuropein (OE), is known for its potential applications as an antimicrobial agent in the treatment of some common olive tree diseases [36]. Remarkably, olive oil has been revealed a positive effect on human health, e.g., reducing the incidence of Alzheimer's disease [37]. Apart from the fruits, olive leaves are also rich in OE, which is an antioxidant with strong anticancer properties [38–40]. Furthermore, the phenolic extract of olive leaves could be used in various industrial applications, including food additives and nutraceuticals [41]. It is well-known that the B3 TFs play important roles in both vegetative and reproductive developments in plants. Previous studies have shown that some B3 genes played a crucial role in the oil biosynthesis [42–44]. For example, in *Arabidopsis*, *LEC2* gene can increase the expression of fatty acid elongase 1 (FAE1), thus inducing the accumulation of triacylglycerols [42]. The mutant *BnFUSCA3* in *Brassica napus*, a gene from LAV subfamily, showed increased levels of linoleic acid, suggesting the important role of *FUSCA3* in the oil biosynthesis [43]. However, the molecular functions of B3 TFs in olive development and oil biosynthesis remain unknown.

In this study, we identified a total of 200 B3 genes in olive genome based on the transcriptomic analysis and further characterized their gene structure, motif composition, and chromosomal locations. The genomic duplication and evolutionary events in the B3 genes were explored among olive, *Arabidopsis*, and rice to investigate their syntenic and gene expansion patterns. Furthermore, the expression patterns of the B3 genes in different tissues (i.e., young and old leaves, pedicels, stems, and fruits) were revealed by the RNA-seq analysis. The RNA-seq and the quantitative real-time polymerase chain reaction (qRT-PCR) analysis of two olive cultivars with significant differences in oil content (i.e., high and low) were performed to investigate the potential factors involved in the regulation of olive oil biosynthesis. Our study provided novel evidence to facilitate the further functional explorations of B3 genes in the oil biosynthesis in olive.

## 2. Method and Materials

### 2.1. Identification of B3 Genes in Olive Genome

Total protein sequences of the olive genome were downloaded from the National Genomics Data Center (NGDC; accession number PRJCA003222). The hidden Markov model (HMM) of the B3 DNA-binding domain (PF02362) was downloaded from the Pfam database (http://pfam.xfam.org/family/PF02362/) with the candidate genes (i.e., *OeB3*) with a threshold of *e* − value < 0.01 detected using the HMMER program (http://hmmer.org/). The conserved domains of all candidate genes were confirmed using the CD-Search (https://www.ncbi.nlm.nih.gov/Structure/cdd/wrpsb.cgi) and InterProScan software (http://www.ebi.ac.uk/interpro/). The theoretical isoelectric points and the molecular weights of olive B3 (*OeB3*) genes were predicted using the ExPASY server (https://web.expasy.org/compute_pi/).

### 2.2. Phylogenetic and Gene Structure Analyses of Olive B3 Genes

Multiple sequence alignments of B3 proteins from olive, *Arabidopsis*, and rice were performed by MAFFT with default parameters [45] and then used as a query of IQ-TREE for phylogenetic analysis with the best model JTT+R4 [46]. Classification of *OeB3* genes was performed based on *AtB3* and *OsB3* genes as previously reported [6]. The sequences of *AtB3* and *OsB3* genes were downloaded from Phytozome 12.1.6 (https://phytozome.jgi.doe.gov/pz/portal.html/). The gene structure was retrieved from the olive genome at the NGDC (accession PRJCA003222) and visualized by TBtools [47]. The conserved motifs were predicted by the online tool MEME (https://meme-suite.org/meme/tools/meme/) with the number of motifs set to 20.

### 2.3. Chromosomal Locations and Syntenic Analysis of the Olive B3 Genes

The chromosomal locations of *OeB3* genes were determined based on the previous study of the olive genome [48] and were visualized using TBtools [47]. The tandem and segmental duplications of *OeB3* genes were identified by the Multiple Collinearity Scan toolkit (MCScanX) [49]. The diagrams of syntenic analysis were plotted using TBtools [47]. The collinearity and syntenic blocks among olive, *Arabidopsis*, and rice were characterized by MCScanX [49]. The alignments of duplicated gene pairs were performed by Para2AT [50], with the nonsynonymous/synonymous substitution (Ka/Ks) ratios calculated by KaKs_Calculator 2.0 [51].

### 2.4. Prediction of *cis*-Elements of OeB3 Genes

To predict the *cis*-acting regulatory elements of *OeB3* genes, the 2 kb sequence in the promoter region upstream of the start codon of each *OeB3* gene was extracted, and the potential *cis*-acting regulatory elements of *OeB3* genes were predicted by PlantCARE online tools [52]. The number of plant hormone-related elements was visualized using the “pheatmap” R package (https://cran.r-project.org/web/packages/pheatmap/index.html/).

### 2.5. Expression Patterns of *OeB3* Genes in Different Tissues Involved in Oil Development

To explore the transcriptional regulation of *OeB3* genes in different tissues of olive, including new and old leaves, pedicel, stem, and fruit, the DNA sequences of the *OeB3* genes of these tissues were collected from the National Center for Biotechnology Information (NCBI; https://www.ncbi.nlm.nih.gov/sra/) database, with the fragments per kilobase per million mapped fragment (FPKM) values of *OeB3* genes transformed for normalization and visualized in a circular heatmap using the “circlize” R package [53]. Transcriptomic data of new and old leaves and fruits of olive were retrieved from the NCBI database (BioProject accession PRJNA596876) and those of stems and pedicels downloaded from the NCBI database (accession PRJNA350601). To investigate the functions of *OeB3* genes in oil biosynthesis, the FPKM values of the *OeB3* genes based on the RNA-seq analysis in two olive cultivars “JZ” and “KLD” with high and low oil contents, respectively, were extracted from the NCBI database (accession PRJNA816306) and normalized to generate a circular heatmap using the “circlize” R package [53]. A total of 16 *OeB3* genes were randomly selected in four B3 gene families for relative expression analysis to validate the relative transcript levels revealed by the RNA-seq analysis in these two olive cultivars, with the expression levels calculated using the 2^−*ΔΔ*Ct^ method and gene *AF28* used as the internal reference. The fruit samples were obtained from the Liangshan Zhongze New Technology Development Co., Ltd. (Xichang, China). The primers used for gene expression of the *OeB3* genes in the relative expression analysis are provided in Table S1.

## 3. Results

### 3.1. Identification and Characteristics of *OeB3* Genes

**Figure 1:**
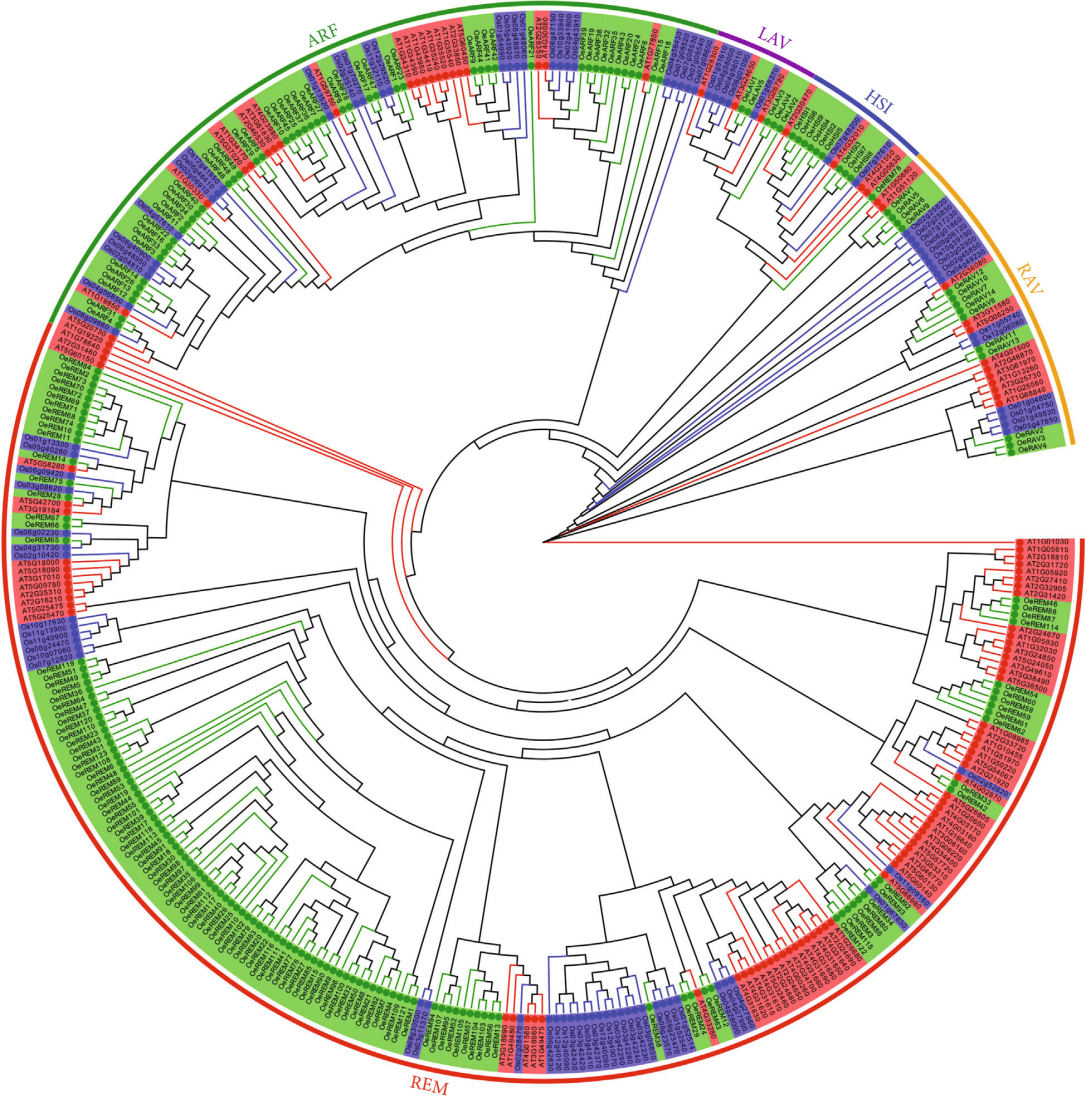
Phylogenetic analysis of *OeB3* genes from *Arabidopsis*, rice, and olive. The maximum likelihood tree is constructed based on a total of 404 B3 protein sequences of *Arabidopsis* (115 highlighted in red), rice (89 presented in blue), and olive (200 displayed in green) revealed in five B3 families, i.e., REM, RAV, LAV, HSI, and ARF.

A total of 200 B3 candidate genes were detected in olive genome based on the HMMER and BLAST with the length of the olive B3 proteins ranging from 99 to 1099 amino acids (average of 396 amino acids) and the molecular weights ranging from 11.83 to 121.85 kDa. The isoelectric point (pI) analysis showed that all these olive B3 proteins were hydrophobic with the maximum pI value of 10.18. A total of 404 conserved B3 proteins and homologous B3 protein sequences from *Arabidopsis*, rice, and olive were used to construct the maximum likelihood trees to explore their phylogenetic relationships (Figure 1). The results showed that the 200 OeB3 proteins were clustered into five clades (i.e., families), including REM, ARF, RAV, HSI, and LAV. The REM clade was the largest family containing a total of 123 OeB3 proteins, while the ARF family was composed of 49 OeB3 proteins, whereas the RAV, HSI, and LAV families consisted of 14, 9, and 5 OeB3 proteins, respectively (Table S2).

### 3.2. Gene Structure and Motif Analyses of OeB3s

**Figure 2:**
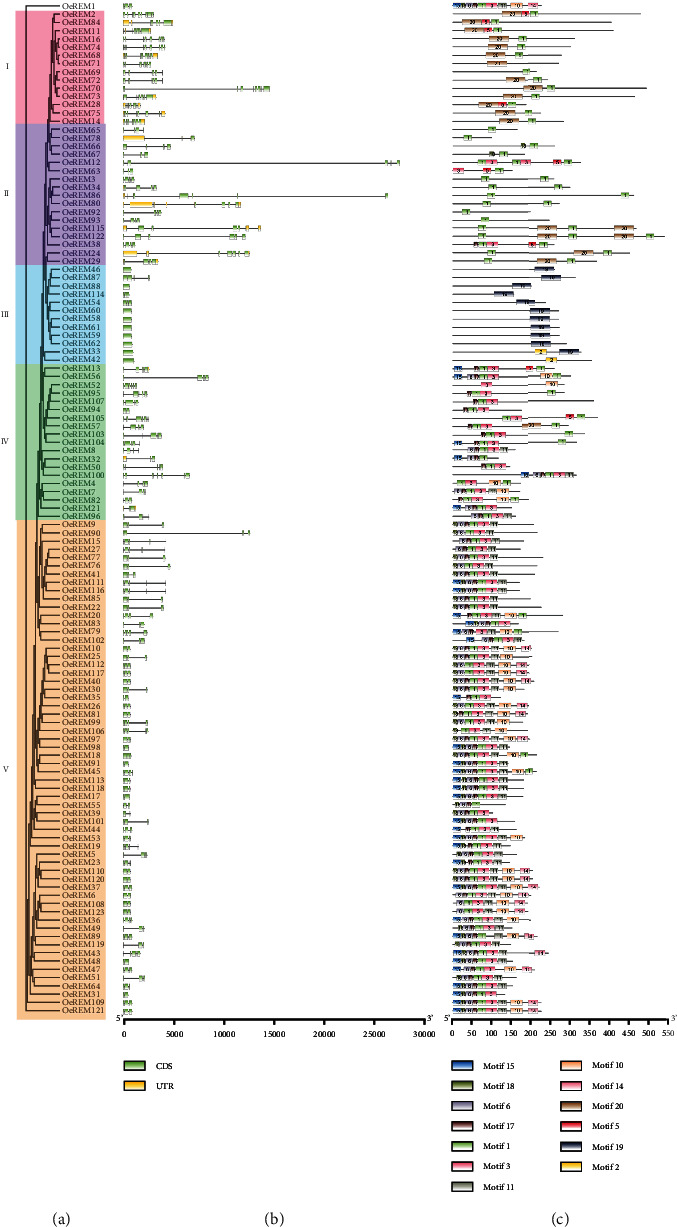
Structure and motif analyses of the *OeB3* genes in the REM family in olive. (a) Phylogenetic analysis and classification of a total of 123 *OeB3* genes revealed in subgroups I to V. (b) Gene structure analysis. Yellow and light green boxes represent UTR and CDS, respectively. The black lines indicate the introns. (c) Motif analysis. A total of 20 motifs are displayed and plotted with different colors and motif IDs.

**Figure 3:**
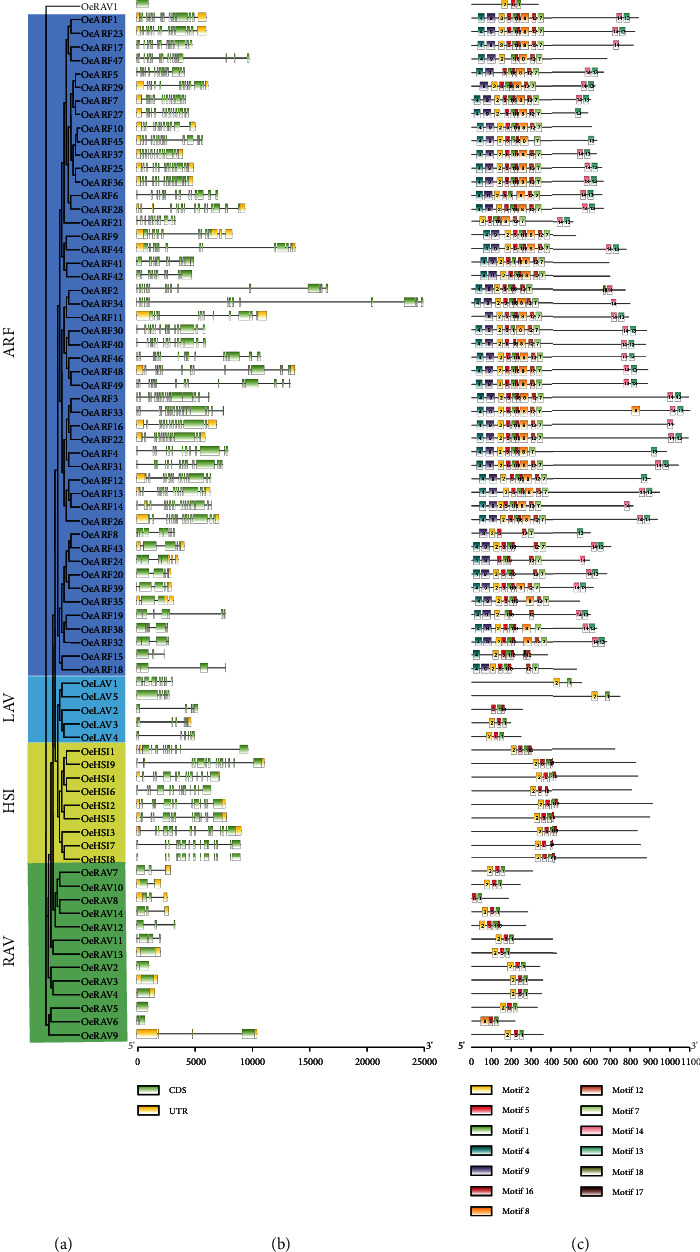
Structure and motif analyses of the *OeB3* genes in the ARF, LAV, HSI, and RAV families in olive. (a) Phylogenetic analysis and classification of a total of 77 *OeB3* genes revealed in four families. (b) Gene structure analysis. Yellow boxes represent UTR, light green boxes represent CDS, and black lines indicate the introns. (c) Motif analysis. A total of 20 motifs are displayed and plotted with different colors and motif IDs.

To investigate the gene structure and conserved motifs of *OeB3* genes, two separate phylogenetic trees were constructed based on the REM family and other four families (RAV, HSI, LAV, and ARF) with the maximum likelihood method, respectively, due to the uneven distributions of the *OeB3* genes in these families. The results showed that among the 123 *OeB3* genes revealed in five subgroups in the REM family, most contained 3–5 coding sequences (CDS) and 10 genes had only 1 CDS encoding a short protein (Figure 2). In subgroup I, the genes contained 7–9 CDS with both motif 1 and motif 20 conserved, except for the absence of motif 20 in *OeREM69*. The REM genes in subgroup II contained 3–5 CDS with multiple copies of motif 1 among the proteins. It was noted that motif 19 was only found in subgroup III with a short protein. Subgroups IV and V were similar in motif composition with the conserved motif sequence 18-6-17-1-3-11. Subgroups IV and V contained 4–5 CDS and 2–3 CDS, respectively. As shown in Figure 3, most *OeB3* genes of the ARF family contained more than 10 CDS, and the motifs were highly conserved with the motif sequences 4-9-2-5-1-16-8-12-7 and 14-13. In the LAV family, two *OeB3* genes were annotated with motifs 1 and 2, whereas the remaining members of LAV family were conserved in motifs 1 and 5. The *OeB3* genes of the HSI family were also conserved with the motif sequence 2-5-1-16, whereas the RAV family was conserved with the motif sequence 2-5-1. These variations in the motif patterns in these B3 families implied the diverse functions of the *OeB3* genes.

### 3.3. Chromosomal Distribution and Tandem Duplications of *OeB3* Genes

**Figure 4:**
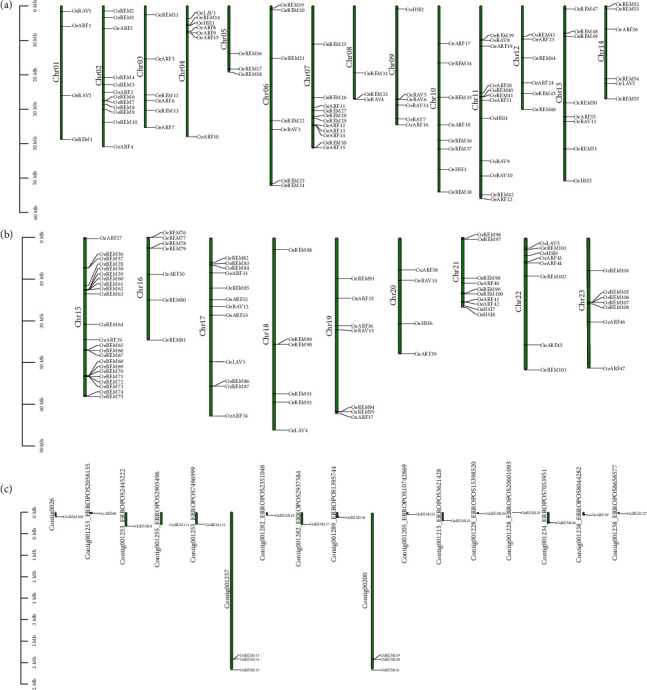
The chromosomal distribution of B3 genes based on the olive genome. The green bars represent different chromosomes and contigs. (a) Chr01 to Chr14. (b) Chr15 to Chr23. (c) 17 contigs with *OeB3* genes.

The results of the chromosomal distribution and tandem duplication analyses showed that the *OeB3* genes were distributed widely on the chromosomes with uneven patterns. Specifically, a total of 21 *OeB3* genes were mapped onto the Chr15, followed by 12 *OeB3* genes on Chr02, Chr11, and Chr17, respectively, while Chr05 and Chr08 each contained a minimum of three *OeB3* genes (Figure 4). A total of 15 contigs were detected with the presence of *OeB3* genes, with the maximum number of 3 genes in both Contig001257 and Contig00200.

**Figure 5:**
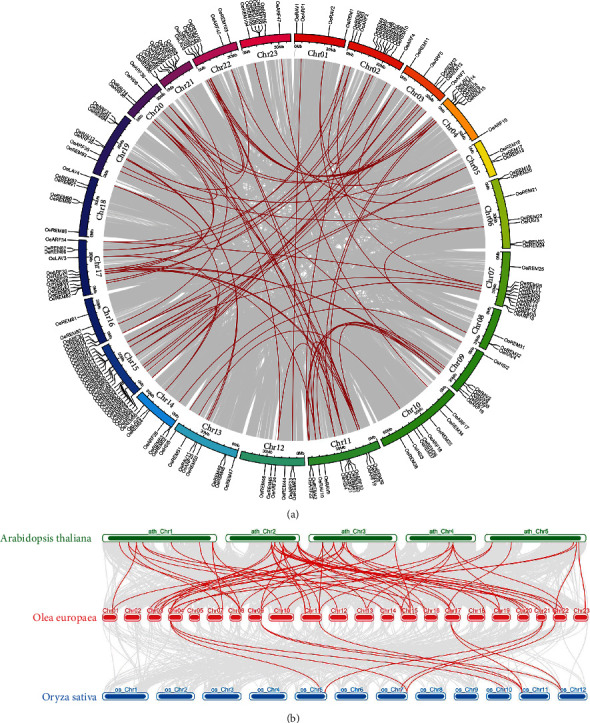
Gene duplications in olive and syntenic blocks between olive and both *Arabidopsis* and rice. (a) Gene duplications in olive. The red lines represent homologous genes of *OeB3* genes (i.e., duplicated genes). The grey lines represent the homologous genes with the *OeB3* genes as the background. Different chromosomes are represented by the blocks in different colors. (b) Syntenic analysis of olive, *Arabidopsis*, and rice. The grey lines represent collinear blocks within all three species. Red lines represent the syntenic B3 gene pairs between olive and either *Arabidopsis* or rice.

To further investigate the uneven distribution patterns of *OeB3* genes on chromosomes, we performed the collinearity and tandem duplication analyses based on the olive genome. The results showed that a total of 87 segmental duplication events in the olive genome were identified, while the syntenic blocks of *OeB3* genes were detected in the chromosomes and eight contigs (Figure 5(a) and Table S3). In particular, 10 tandem duplication events were located in Chr07, Chr15, Chr16, Chr19, and Chr23, with the maximum number of tandem duplications (6) identified in Chr15 (Table S3). These results indicated that the segmental and tandem duplications contributed largely to the expansion of *OeB3* genes. The Ka/Ks ratios of a total of 97 duplicated gene pairs were calculated to assess the selection pressure of *OeB3* genes. The results showed that the Ka/Ks ratios of a total of 70 *OeB3* gene pairs were less than 1, whereas the Ka/Ks ratios of 23 gene pairs were greater than 1. The high percentage of gene pairs with the Ka/Ks ratio < 1 demonstrated that the purifying selection had a strong effect on *OeB3* genes during the evolution of olive.

A comparative syntenic analysis was performed among olive, *Arabidopsis*, and rice to further investigate the evolutionary relationships of B3 genes among these three species (Figure 5(b)). The results showed that a total of 68 corresponding gene pairs of the *OeB3* genes were identified in *Arabidopsis*, which was six times higher than that in rice with 11 gene pairs (Table S4). This large variation in the number of homologous genes indicated that the divergence of B3 genes between olive and *Arabidopsis* occurred after the divergence of B3 genes between rice and the common ancestor of dicotyledons. The maximum number of syntenic blocks between olive and *Arabidopsis* was up to 64, whereas none of the syntenic blocks between olive and rice contained more than 30 genes, indicating that *OeB3* genes had the similar functions as those of the *Arabidopsis* orthologs (Table S5).

### 3.4. Prediction of *cis*-Elements in the Promoter of *OeB3* Genes of Olive

**Figure 6:**
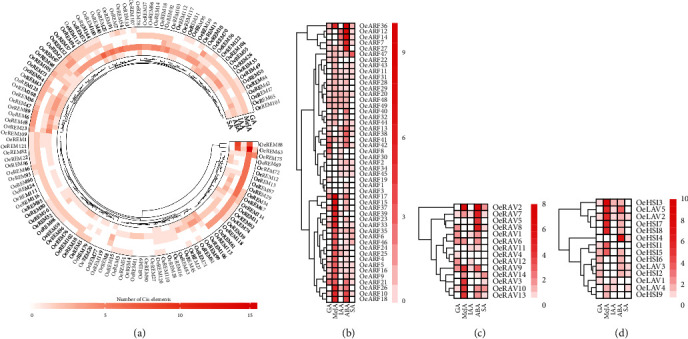
The *cis*-element analysis of *OeB3* genes in olive in the families of (a) REM, (b) ARF, (c) RAV, and (d) combination of LAV and HSI. The heatmap value represents the number of *cis*-elements identified in the *OeB3* genes.

The *cis*-acting regulatory elements in the promoter play important roles in the regulation of downstream gene expression by the TFs. To investigate the biological functions of *OeB3* genes, a 2 kb upstream sequence from *OeB3* genes was extracted to predict the *cis*-acting regulatory elements based on the PlantCARE database. The results showed that a total of 113, 128, 96, 172, and 99 *OeB3* genes with *cis*-elements were related to gibberellin (GA), methyl jasmonate (MeJA), auxin (IAA), abscisic acid (ABA), and salicylic acid (SA), respectively (Figure 6 and Table S6). For example, *OeREM63* contained a maximum number of 16 MeJA response elements and 8 ABA response elements, suggesting that *OeREM63* was involved in the molecular response to MeJA and ABA in olive (Figure 6(a)). In the ARF family, 11, 9, and 9 ABA response elements were detected in *OeARF36*, *OeARF1*2, and *OeARF1*4, respectively, whereas no ABA response elements were detected in *OeARF8*, *OeARF1*9, *OeARF22*, *OeARF30*, and *OeARF39* (Figure 6(b)). It was noted that none of the hormone-related elements were detected in *OeREM119*.

### 3.5. Tissue-Specific Expression Patterns of *OeB3* Genes

**Figure 7:**
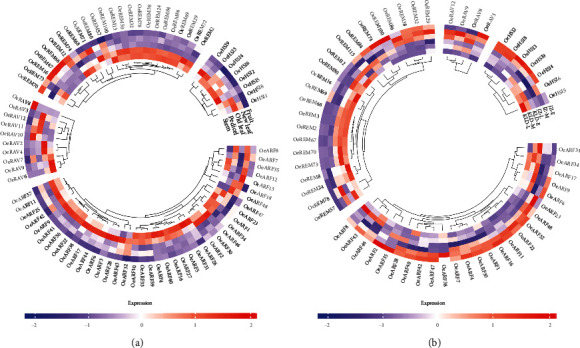
Expression patterns of *OeB3* genes in olive. (a) Heatmap of expression profiles of *OeB3* genes in different tissues of olive, including stem, pedicel, young leaf, old leaf, and fruit. (b) Expression profiles of *OeB3* genes in two fruit cultivars of olive, i.e., JZ (low oil content) and KLD (high oil content), during the early (E), middle (M), and late (L) stages of the mature fruits.

To explore the tissue-specific expression profiles of *OeB3* genes in olive genome, the *OeB3* genes with high expression levels were identified in different tissues, including young leaf, old leaf, stem, pedicel, and fruit, based on the transcriptomic data previously published (Figure 7(a)). The results showed that most *OeB3* genes were highly expressed in stem and pedicel, while some *OeB3* genes displayed a tissue-specific expression pattern. For example, in the REM family, a total of six genes (i.e., *OeREM12*, *OeREM16*, *OeREM66*, *OeREM67*, *OeREM70*, and *OeREM73*) were expressed at higher levels in the stem than those in other tissues, whereas a group of 12 genes (i.e., *OeREM2*, *OeREM2*4, *OeREM2*9, *OeREM3*2, *OeREM3*8, *OeREM50*, *OeREM69*, *OeREM72*, *OeREM78*, *OeREM84*, *OeREM86*, and *OeREM100*) showed the pedicel- and stem-specific expression patterns. In the RAV family, *OeRAV8* was detected with a high expression level in the stem, whereas *OeRAV6*, *OeRAV7*, and *OeRAV9* were highly expressed in the fruit. Similarly, most *OeB3* genes in both HSI and ARF families were highly expressed in the pedicel and stem, suggesting that these genes were involved in plant development, though a few genes in the ARF family were involved in fruit development, such as *OeARF7*, *OeARF13*, and *OeARF34*. It was noted that several duplicated gene pairs showed evident divergence on the expression profiles (Table S7). For example, *OeRAV7* was expressed in young leaf, pedicel, and stem, whereas its duplicate gene (i.e., *OeRAV8*) exhibited a high expression level in fruit. In addition, two duplicated genes (*OeRAV12* and *OeRAV14*) showed a predominant pattern in old leaf and young leaf, respectively (Table S7). Overall, these results suggested that the *OeB3* genes evolved diverse functions during their evolution.

### 3.6. Spatial and Temporal Expression Patterns of *OeB3* Genes during the Olive Fruit Development

**Figure 8:**
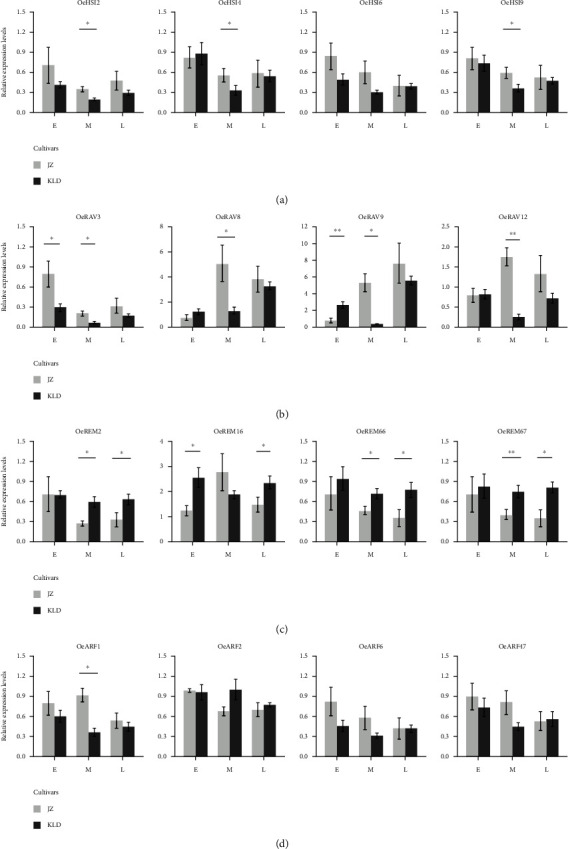
The quantitative real-time PCR (qRT-PCR) analysis of *OeB3* genes at the early (E), middle (M), and late (L) stages of mature fruits in two fruit cultivars (i.e., “KLD” with high oil content and “JZ” with low oil content). (a) Four *OeB3* genes in the HSI family. (b) Four *OeB3* genes in the RAV family. (c) Four *OeB3* genes in the REM family. (d) Four *OeB3* genes in the ARF family. Error bars represent the standard deviation of three independent experiments. Symbols “^∗^” and “^∗∗^” represent the significant differences at *P* < 0.05 and *P* < 0.01, respectively.

To illustrate the functions of *OeB3* genes in oil biosynthesis, the RNA-seq data of two fruit cultivars of olive with significant difference in oil content, i.e., “KLD” with high oil content and “JZ” with low oil content, were explored to evaluate the expression levels of *OeB3* genes during the early (E), middle (M), and late (L) developmental stages of mature fruits (Figure 7(b)). The results showed that three members of the HSI family (*OeHSI2*, *OeHSI3*, and *OeHSI8*) were highly expressed in JZ-E and JZ-M and one member (*OeHSI5*) was expressed specifically in both KLD-M and KLD-L, whereas three members (*OeHSI4*, *OeHSI6*, and *OeHSI9*) exhibited a mixed expression patterns in JZ and KLD. Most of the *OeB3* genes of the RAV family showed high expression levels in JZ-M and JZ-L, whereas only one gene (*OeRAV3*) was highly expressed in JZ-E (Figure 7(b)). Notably, most *OeB3* genes of the REM family showed higher expression levels in KLD than those in JZ, suggesting that the genes in the REM family played vital functions in facilitating the oil biosynthesis in olive (Figure 7(b)). For example, a group of six genes (i.e., *OeREM2*, *OeREM3*, *OeREM66*, *OeREM67*, *OeREM70*, and *OeREM73*) was highly expressed in KLD-E, KLD-M, and KLD-L, whereas *OeREM74*, *OeREM84*, and *OeREM100* were expressed specifically in JZ-E. Interestingly, most *OeB3* genes of the ARF family were expressed at high levels in JZ, showing the reversed expression patterns in comparison to those of the genes in the REM family (Figure 7(b)). For example, *OeARF1* and *OeARF47* showed high expression levels in JZ-E, JZ-M, and JZ-L, and the relative expressions of *OeARF1* and *OeARF47* showed a similar trend in JZ-E and JZ-L. These expression patterns revealed by the RNA-seq analysis were largely verified by the relative expression analysis. For example, the results of relative expression analysis showed that the high expression levels of four HSI members (*OeHSI2*, *OeHSI4*, *OeHSI6*, and *OeHSI9*) were verified by the relative expression analysis in JZ-E, while *OeHSI2*, *OeHSI4*, and *OeHSI9* were more significantly upregulated (*P* < 0.05) in JZ-M than in KLD-M (Figure 8(a)). Furthermore, *OeREM2*, *OeREM66*, and *OeREM67* were more significantly upregulated (*P* < 0.05) in KLD than in JZ at both M and L stages of the mature fruits, whereas *OeREM16* was highly expressed (*P* < 0.05) in KLD than in JZ at E and L stages (Figure 8(c)). Moreover, *OeARF1* was more highly expressed in JZ than in KLD, in particular, with significant difference (*P* < 0.01) at M stage of mature fruits (Figure 8(d)). These diverse expression patterns of *OeB3* genes suggested that these genes played different functions in oil biosynthesis in olive.

## 4. Discussion

As one of the largest and most widely distributed plant-specific superfamilies, the B3 TFs are well-known to have varied regulatory functions in diverse types of developmental processes in plants [6, 54]. Although the B3 genes have been identified in several species of crop plants, the comprehensive molecular characterizations of the B3 genes at the genomic level in olive are still lacking. In our study, a total of 200 candidate B3 genes, accounting for 0.37% of the total 53,517 predicted genes in olive, were identified containing the typical B3 protein domain based on a comprehensive set of well-established gene prediction methods. The proportion of *OeB3* genes in the total predicted genes in olive was lower than that of the *AtB3* genes (0.43%) in *Arabidopsis* but higher than those of the rice *OsB3* genes (0.16%) and sweet orange *CsB3* genes (0.24%) [55]. The phylogenetic analysis of B3 genes among olive, rice, and *Arabidopsis* indicated that the *OeB3* genes in olive were classified into five families corresponding to the homologous B3 genes in *Arabidopsis* and rice [6], including REM, ARF, RAV, HSI, and LAV. These groups were also supported by the shared gene structure and motif compositions among the genes in each family. Our study showed that the REM family was the largest group with a total of 123 B3 members identified in olive, which was similar to those of B3 members in *Arabidopsis* and rice. Notably, the members in the REM family showed a large variation in their B3 domains. For example, our results showed that the protein length was increased in the first B3 domain of *OeREM70* and *OeREM73*, whereas the length of protein was increased in the second B3 domain of *OeREM84* (Figure S1). Furthermore, the AP2 domain was absent in some RAV proteins [6, 56]. In our study, the members in the RAV family were highly conserved in their B3 domains, while four genes (i.e., *OeRAV6*, *OeRAV8*, *OeRAV12*, and *OeRAV14*) lacked the typical AP2 domain (Figure S2). This observation was consistent with that reported in pineapple [57], suggesting the conserved structure in the members of the RAV family.

The results of the gene duplication analysis in the olive genome revealed that the gene duplication played an essential role in the evolution of *OeB3* genes in olive, as suggested by the large variation in the copy number of the *OeB3* genes in the five families. Among the total of 87 segmental duplication events and 10 tandem duplication events, a total of 33 duplication events were identified in the REM family (Table S8), widely distributed in the chromosomes. The results of chromosomal location analysis showed that the *OeB3* genes in the REM family were clustered tightly in the chromosomes. These results were consistent with those reported in *Arabidopsis*, rice, sweet orange, and tobacco [15, 55]. Furthermore, the results of the Ka/Ks ratio analysis of homologous genes showed that the Ka/Ks ratios were less than 1 in a total of 70 gene pairs, whereas the Ka/Ks ratios were greater than 1 in the remaining 23 gene pairs, suggesting the positive selection on the *OeB3* genes during their evolution.

Studies showed that the genomic synteny between *Arabidopsis* and rice could be used to predict the gene functions in nonmodel plants [58]. In our study, a total of 68 gene pairs were identified as segmental duplication events between olive and *Arabidopsis*, while there were a total of 11 gene pairs between olive and rice, suggesting that these two groups of gene pairs were originated from different ancestors (Table S4). The large number of syntenic blocks between *Arabidopsis* and olive suggested that the B3 genes were originated before the divergence of *Arabidopsis* and olive. These results were consistent with those reported previously [48].

The functions of *OeB3* genes were further explored based on their expression levels in different tissues, including young leaf, old leaf, stem, pedicel, and fruit, at developmental stages based on transcriptomic analysis. Notably, no members of the LAV family were expressed in these tissues, probably due to the unique expression patterns of the members in the LAV family. Further studies are needed to clarify the expression patterns of *OeB3* genes in the LAV family. Our results showed that most *OeB3* genes showed stem- and pedicel-specific expression patterns, indicating that these *OeB3* genes played a crucial role in the vegetative growth and development in olive (Figure 7). For example, a total of 11 members of the REM family (i.e., *OeREM2*, *OeREM2*4, *OeREM2*9, *OeREM3*2, *OeREM3*8, *OeREM50*, *OeREM69*, *OeREM72*, *OeREM78*, *OeREM84*, and *OeREM86*) and three members of the HSI family (*OeHSI3*, *OeHSI5*, and *OeHSI9*) were expressed at high levels in stem and pedicel compared with the other three tissues. Studies showed that genes in the ARF family regulated the expression of auxin response genes to influence the auxin production by binding to TFTCTC auxin response elements (AuxRE) on the promoter region, ultimately downregulating the expression of senescence-associated genes (SAGs) and delaying leaf senescence [59, 60]. In our study, a total of four ARF genes (*OeARF1*8, *OeARF23*, *OeARF47*, and *OeARF48*) were highly expressed in old leaf, indicating that these genes were involved in leaf senescence of olive. Furthermore, five genes in the HSI family (*OeHSI2*, *OeHSI4*, *OeHSI6*, *OeHSI8*, and *OeHSI11*) and four genes in the RAV family (*OeRAV3*, *OeRAV10*, *OeRAV11*, and *OeRAV12*) were expressed in old leaf, whereas only a few genes, such as *OeRAV8*, were highly expressed in fruit, suggesting the tissue-specific expression pattern of these genes during fruit development in olive.

The molecular functions of *OeB3* genes involved in the oil biosynthesis in olive were investigated based on two fruit cultivars of olive, i.e., KLD and JZ with high and low oil contents, respectively. The potential B3 genes regulating the oil biosynthesis were identified based on the transcriptomic data of these two cultivars at the early (E), middle (M), and late (L) stages of mature fruits. Our results showed that most *OeB3* genes of the REM family were expressed at high levels in KLD, whereas most *OeB3* genes in the ARF family were highly expressed in JZ (Figure 7). These results suggested that the *OeB3* genes in REM and ARF families regulated the oil biosynthesis with different molecular mechanisms. Furthermore, some *OeB3* genes were only expressed at a specific stage, suggesting their essential roles in oil biosynthesis. For instance, one of the four members of the RAV family (i.e., *OeRAV3*) showed high expression levels in JZ-E and JZ-M, whereas the remaining three members of the RAV family (*OeRAV8*, *OeRAV9*, and *OeRAV12*) were expressed at both JZ-M and JZ-L. Furthermore, the *OeB3* genes in the HSI family showed varied expression patterns, indicating the diverse functions of the HSI family in oil biosynthesis. Moreover, the expression patterns revealed by the transcriptomic analysis were verified by the relative expression analysis. Previous studies show that phytohormones participated in oil biosynthesis, especially ABA and GA [61, 62]. Using the information of *cis*-elements related to ABA and GA, we could further determine several potential B3 genes which may be involved in oil biosynthesis. For example, *OeREM67*, which contained 3 ABA elements and 2 GA elements, showed expression level two times higher in KLD than that in JZ, suggesting that this gene may be involved in oil biosynthesis via ABA and/or GA pathways. The gene *OeREM38*, containing 2 GA elements, was only expressed in JZ, indicating that it may be regulated by GA pathway. Overall, these results suggested that these *OeB3* genes played a crucial role in oil biosynthesis in olive.

## Data Availability

Data are available within the manuscript.

## Abbreviations

GA: Gibberellin
MeJA: Methyl jasmonate
IAA: Indole-3-acetic acid
ABA: Abscisic acid
SA: Salicylic acid
qRT-PCR: Quantitative real-time PCR
RNA-seq: RNA-sequencing
NGDC: National Genomics Data Center
CDS: Coding sequences.

## Supplementary Materials

Supplementary Materials Table S1: the primers used in the qRT-PCR experiments in this study. Table S2: the overview of B3 superfamily in olive. Table S3: duplicated genes of B3 superfamily identified in olive. Table S4: the duplicated genes of B3 superfamily between olive and *Arabidopsis* and between olive and rice. Table S5: the largest syntenic blocks between olive and *Arabidopsis* and between olive and rice. Table S6: hormone-related *cis*-elements identified in the B3 superfamily in olive. Table S7: the expression levels of duplicated genes of B3 superfamily in olive. Table S8: the Ka/Ks ratios of duplicated genes of B3 superfamily in olive. Figure S1: the multiple alignment of the B3 proteins in the REM family of B3 genes in olive showing the two domains (a, b) located in the REM family. Figure S2: the multiple alignment of the B3 proteins in the RAV family of B3 genes in olive showing the AP2 and B3 domains.
