# Supplementary material for: Genome-Wide Analysis of the Molecular Functions of B3 Superfamily in Oil Biosynthesis in Olive (Olea europaea L.)
Source: Biomed Res Int. 2023 Feb 14;2023:6051511. doi: 10.1155/2023/6051511 (PMC9943606; doi:10.1155/2023/6051511)
Supplement: Supplementary Materials — Table S1: the primers used in the qRT-PCR experiments in this study. Table S2: the overview of B3 superfamily in olive. Table S3: duplicated genes of B3 superfamily identified in olive. Table S4: the duplicated genes of B3 superfamily between olive and Arabidopsis and between olive and rice. Table S5: the largest syntenic blocks between olive and Arabidopsis and between olive and rice. Table S6: hormone-related cis-elements identified in the B3 superfamily in olive. Table S7: the expression levels of duplicated genes of B3 superfamily in olive. Table S8: the Ka/Ks ratios of duplicated genes of B3 superfamily in olive. Figure S1: the multiple alignment of the B3 proteins in the REM family of B3 genes in olive showing the two domains (a, b) located in the REM family. Figure S2: the multiple alignment of the B3 proteins in the RAV family of B3 genes in olive showing the AP2 and B3 domains. [file 6051511.f1.zip › Suply-Table(S1-S8).docx]

| **Table S1. The primers used in the qRT-PCR experiments in this study** | | |
| --- | --- | --- |
| gene | Forward Primers (5'->3') | Reverse Primers |
| AF28 | AATGAAGTCTGTCTCTCCTTTGG | AAGGGAAATCCCATCAACG |
| OeARF1 | GGGAGCTGAATATGGTTACTCT | AGTTAAGTTGGGATGGAGTACC |
| OeARF2 | AGAAGCTGGGAGAACAAGA | TCAGTATTCAAGCGACCCTA |
| OeARF6 | TCTATACCACTGAGGAAGC | CGAGGATTGTTCTTCAGAG |
| OeARF47 | AAGATCGCCACCTACTTAT | AGTATTTGCCCACCTGAT |
| OeHSI2 | TTTTGGTCATCTCTCTCGGTCTA | CCCAAGAGTTCATAGCAACTTCA |
| OeHSI4 | CGACCTTAAATCAGAAGACGATGA | GTCCATTGGGTTTGTGTCCTTA |
| OeHSI6 | GCAGGTTTACAGAAGAACGAAATC | CATCAGCGATATGTTCACTACTCT |
| OeHSI9 | ACAGTCCTATAGCTTGTACTATGG | GTAGGTGGAAGAACATAACCAAC |
| OeRAV3 | CGGACAATCAACTCTACATC | AGTCTTACCATCGGAATAGG |
| OeRAV8 | TTTCTCAGGAGATGTCAACCA | GGAATTAGCCAATCCTGGAAG |
| OeRAV9 | GGAGTGCAAATTACTTGAGGAAGA | CCAGAGATAACAGGAAACGTGTAG |
| OeRAV12 | GTCCAAGAATCTTATGTTGCTCAG | GTAACCAGAATGTCTCCACTCA |
| OeREM2 | AGCTCGAACTGAAGATGA | ATCTCTCCGCCTTTGATT |
| OeREM16 | GGGTTAATTTGGATCTCGTGAAG | TTTGCCTTTACTTTGACTGCTC |
| OeREM66 | AACAATCTAGTGGTTGGTGATG | CTTGCAGTTCTGGTGGAATT |
| OeREM67 | CCTGGAAAGCAGTTCTGTAAA | CGATAATGATTGCTTGACGTTC |
| OeREM70 | GTTCTCGGTTAAGCTATCTTGTAA | AATCTGAATCTCTTCTTCTCTGC |

**Table S2. The overview of B3 superfamily in olive**

| **Gene Name** | **Gene ID** | **Group** | **Protein Length (aa)** | **CDS Length (bp)** | **MW (kDa)** | **PI** | **Chromosome** | **Location** |
| --- | --- | --- | --- | --- | --- | --- | --- | --- |
| OeRAV1 | GWHGAOPM000109 | RAV | 333 | 1002 | 38.49535 | 5.84 | Chr01 | 1676512-1677513 |
| OeARF1 | GWHGAOPM000403 | ARF | 841 | 2513 | 93.99579 | 6.08 | Chr01 | 5940750-5946767 |
| OeRAV2 | GWHGAOPM001565 | RAV | 342 | 1028 | 38.30118 | 8.55 | Chr01 | 26076628-26077688 |
| OeREM1 | GWHGAOPM002123 | REM | 226 | 679 | 26.79799 | 5.72 | Chr01 | 38864180-38865038 |
| OeREM2 | GWHGAOPM002258 | REM | 478 | 1429 | 53.67834 | 8.22 | Chr02 | 1455908-1458881 |
| OeREM3 | GWHGAOPM002359 | REM | 259 | 777 | 29.66687 | 9.46 | Chr02 | 3440748-3441825 |
| OeARF2 | GWHGAOPM002508 | ARF | 774 | 2312 | 86.72741 | 6.69 | Chr02 | 6604000-6620562 |
| OeREM4 | GWHGAOPM002925 | REM | 172 | 516 | 20.33755 | 5.45 | Chr02 | 20868415-20870800 |
| OeREM5 | GWHGAOPM003018 | REM | 162 | 487 | 19.18436 | 5 | Chr02 | 22887538-22889863 |
| OeARF3 | GWHGAOPM003114 | ARF | 1093 | 3267 | 121.245 | 6.28 | Chr02 | 25046699-25052966 |
| OeREM6 | GWHGAOPM003214 | REM | 200 | 601 | 23.42134 | 5.53 | Chr02 | 27495854-27496592 |
| OeREM7 | GWHGAOPM003221 | REM | 170 | 510 | 19.54905 | 5.36 | Chr02 | 27630455-27632667 |
| OeREM8 | GWHGAOPM003280 | REM | 158 | 474 | 18.41399 | 5.74 | Chr02 | 28613515-28615054 |
| OeREM9 | GWHGAOPM003382 | REM | 207 | 621 | 24.20117 | 5.7 | Chr02 | 30095119-30099130 |
| OeREM10 | GWHGAOPM003492 | REM | 201 | 605 | 23.94103 | 5.84 | Chr02 | 33872167-33872866 |
| OeARF4 | GWHGAOPM003954 | ARF | 981 | 2934 | 108.5061 | 5.79 | Chr02 | 40985528-40993428 |
| OeREM11 | GWHGAOPM004396 | REM | 408 | 1223 | 46.24394 | 5.62 | Chr03 | 2537726-2540419 |
| OeARF5 | GWHGAOPM005137 | ARF | 666 | 1988 | 74.72052 | 5.99 | Chr03 | 15368742-15372881 |
| OeREM12 | GWHGAOPM005461 | REM | 326 | 977 | 37.18001 | 6.43 | Chr03 | 25899630-25927101 |
| OeARF6 | GWHGAOPM005523 | ARF | 658 | 1964 | 74.07238 | 5.6 | Chr03 | 27501421-27508444 |
| OeREM13 | GWHGAOPM005686 | REM | 259 | 777 | 30.16772 | 9.59 | Chr03 | 30426073-30428609 |
| OeARF7 | GWHGAOPM006011 | ARF | 600 | 1790 | 67.9878 | 8.21 | Chr03 | 35386676-35390908 |
| OeLAV1 | GWHGAOPM006235 | LAV | 556 | 1662 | 63.11835 | 8.44 | Chr04 | 3643429-3646520 |
| OeREM14 | GWHGAOPM006356 | REM | 283 | 846 | 32.63194 | 9.39 | Chr04 | 5494820-5496940 |
| OeHSI1 | GWHGAOPM006368 | HSI | 722 | 2160 | 79.75524 | 6.85 | Chr04 | 5725008-5734661 |
| OeARF8 | GWHGAOPM006478 | ARF | 600 | 1795 | 66.01565 | 6.3 | Chr04 | 7471687-7474941 |
| OeARF9 | GWHGAOPM006492 | ARF | 526 | 1572 | 58.58021 | 7.59 | Chr04 | 7763318-7771569 |
| OeREM15 | GWHGAOPM006494 | REM | 180 | 539 | 20.97863 | 4.56 | Chr04 | 7821937-7826158 |
| OeARF10 | GWHGAOPM007921 | ARF | 607 | 1812 | 68.50098 | 7.13 | Chr04 | 38013385-38018441 |
| OeREM16 | GWHGAOPM008419 | REM | 310 | 926 | 35.20409 | 9.1 | Chr05 | 13910288-13914335 |
| OeREM17 | GWHGAOPM008667 | REM | 178 | 536 | 20.7495 | 6.22 | Chr05 | 18358591-18359208 |
| OeREM18 | GWHGAOPM008736 | REM | 215 | 646 | 25.31768 | 5.86 | Chr05 | 19297773-19298555 |
| OeREM19 | GWHGAOPM009450 | REM | 146 | 437 | 17.31212 | 5.05 | Chr06 | 611855-613337 |
| OeREM20 | GWHGAOPM009492 | REM | 281 | 843 | 32.3435 | 5.99 | Chr06 | 1209749-1212669 |
| OeREM21 | GWHGAOPM010206 | REM | 149 | 449 | 17.58382 | 5.06 | Chr06 | 15210213-15211459 |
| OeREM22 | GWHGAOPM010882 | REM | 226 | 679 | 26.36432 | 5.07 | Chr06 | 33296566-33300534 |
| OeRAV3 | GWHGAOPM010989 | RAV | 357 | 1074 | 39.7428 | 8.77 | Chr06 | 35903674-35905474 |
| OeREM23 | GWHGAOPM011965 | REM | 144 | 432 | 17.35929 | 5.44 | Chr06 | 51551397-51552127 |
| OeREM24 | GWHGAOPM012017 | REM | 450 | 1348 | 50.98136 | 6.26 | Chr06 | 52194811-52207318 |
| OeREM25 | GWHGAOPM012711 | REM | 203 | 610 | 23.57165 | 6.24 | Chr07 | 11101584-11103898 |
| OeREM26 | GWHGAOPM013363 | REM | 194 | 583 | 22.75065 | 5.14 | Chr07 | 26720835-26721546 |
| OeARF11 | GWHGAOPM013510 | ARF | 792 | 2368 | 89.87738 | 6.33 | Chr07 | 29487417-29498665 |
| OeREM27 | GWHGAOPM013568 | REM | 171 | 512 | 19.66091 | 4.58 | Chr07 | 30428575-30432683 |
| OeREM28 | GWHGAOPM013665 | REM | 186 | 557 | 21.39346 | 9.41 | Chr07 | 32170509-32172208 |
| OeREM29 | GWHGAOPM013703 | REM | 367 | 1101 | 41.75295 | 7.08 | Chr07 | 32690623-32694054 |
| OeARF12 | GWHGAOPM013812 | ARF | 900 | 2689 | 100.0316 | 5.85 | Chr07 | 34528318-34534733 |
| OeARF13 | GWHGAOPM013821 | ARF | 946 | 2828 | 105.2265 | 5.53 | Chr07 | 34764033-34770377 |
| OeARF14 | GWHGAOPM013822 | ARF | 814 | 2430 | 90.28546 | 6.28 | Chr07 | 34795701-34802165 |
| OeREM30 | GWHGAOPM014233 | REM | 181 | 544 | 21.0866 | 5.64 | Chr07 | 41088403-41090744 |
| OeARF15 | GWHGAOPM014242 | ARF | 381 | 1144 | 43.79732 | 9.35 | Chr07 | 41230025-41232404 |
| OeREM31 | GWHGAOPM015141 | REM | 132 | 398 | 15.6926 | 5.94 | Chr08 | 19567714-19568197 |
| OeREM32 | GWHGAOPM015541 | REM | 116 | 350 | 13.27178 | 4.44 | Chr08 | 26820151-26823277 |
| OeRAV4 | GWHGAOPM015562 | RAV | 351 | 1056 | 39.36022 | 8.79 | Chr08 | 27173450-27174991 |
| OeHSI2 | GWHGAOPM015868 | HSI | 910 | 2721 | 100.19 | 6.24 | Chr09 | 1034244-1041879 |
| OeRAV5 | GWHGAOPM016934 | RAV | 328 | 987 | 37.75303 | 9.61 | Chr09 | 27167358-27168344 |
| OeRAV6 | GWHGAOPM016936 | RAV | 217 | 653 | 25.40975 | 9.06 | Chr09 | 27202787-27203503 |
| OeREM33 | GWHGAOPM017025 | REM | 327 | 984 | 37.93309 | 5 | Chr09 | 28889572-28890555 |
| OeRAV7 | GWHGAOPM017295 | RAV | 306 | 919 | 34.83843 | 5.61 | Chr09 | 32883817-32886742 |
| OeARF16 | GWHGAOPM017389 | ARF | 1020 | 3051 | 112.7114 | 6.55 | Chr09 | 34506508-34513410 |
| OeARF17 | GWHGAOPM017781 | ARF | 815 | 2434 | 91.28633 | 6.25 | Chr10 | 10996645-11001425 |
| OeREM34 | GWHGAOPM017983 | REM | 299 | 897 | 33.93224 | 8.75 | Chr10 | 16594086-16597339 |
| OeREM35 | GWHGAOPM018456 | REM | 121 | 365 | 14.22395 | 4.58 | Chr10 | 26627337-26627838 |
| OeARF18 | GWHGAOPM018885 | ARF | 530 | 1590 | 58.04925 | 5.82 | Chr10 | 34589855-34597538 |
| OeREM36 | GWHGAOPM019173 | REM | 199 | 597 | 23.38124 | 6.12 | Chr10 | 39033135-39033989 |
| OeREM37 | GWHGAOPM019360 | REM | 222 | 667 | 26.29571 | 6.08 | Chr10 | 41607900-41608746 |
| OeHSI3 | GWHGAOPM019835 | HSI | 835 | 2495 | 92.52879 | 6.24 | Chr10 | 47560932-47569975 |
| OeREM38 | GWHGAOPM020311 | REM | 259 | 777 | 29.94598 | 6.03 | Chr10 | 54109859-54111017 |
| OeREM39 | GWHGAOPM021658 | REM | 102 | 308 | 11.95939 | 4.69 | Chr11 | 9926100-9926804 |
| OeRAV8 | GWHGAOPM021666 | RAV | 184 | 553 | 21.23012 | 10.18 | Chr11 | 10025238-10027861 |
| OeARF19 | GWHGAOPM021778 | ARF | 599 | 1794 | 66.52591 | 6.21 | Chr11 | 11723660-11731307 |
| OeARF20 | GWHGAOPM022620 | ARF | 681 | 2043 | 75.338 | 8.98 | Chr11 | 25240334-25243296 |
| OeREM40 | GWHGAOPM022677 | REM | 208 | 626 | 24.56574 | 5.25 | Chr11 | 26402468-26403188 |
| OeREM41 | GWHGAOPM022678 | REM | 210 | 631 | 24.54777 | 7.75 | Chr11 | 26408643-26409825 |
| OeARF21 | GWHGAOPM022731 | ARF | 512 | 1529 | 57.41813 | 8.5 | Chr11 | 27415736-27419061 |
| OeHSI4 | GWHGAOPM022959 | HSI | 839 | 2508 | 93.39365 | 7.01 | Chr11 | 32657075-32664250 |
| OeRAV9 | GWHGAOPM023375 | RAV | 359 | 1079 | 40.19768 | 5.91 | Chr11 | 45064012-45074449 |
| OeRAV10 | GWHGAOPM023559 | RAV | 242 | 728 | 27.33955 | 7.77 | Chr11 | 49364771-49366842 |
| OeREM42 | GWHGAOPM023881 | REM | 354 | 1065 | 40.52632 | 4.79 | Chr11 | 54852953-54854017 |
| OeARF22 | GWHGAOPM023961 | ARF | 1090 | 3260 | 120.7552 | 6.42 | Chr11 | 55927771-55933693 |
| OeREM43 | GWHGAOPM024520 | REM | 244 | 731 | 28.80232 | 6.21 | Chr12 | 8864689-8866365 |
| OeARF23 | GWHGAOPM024600 | ARF | 822 | 2456 | 92.31468 | 5.99 | Chr12 | 9757838-9763845 |
| OeREM44 | GWHGAOPM024977 | REM | 161 | 484 | 18.74135 | 6.03 | Chr12 | 15198949-15199808 |
| OeARF24 | GWHGAOPM025413 | ARF | 596 | 1788 | 65.99787 | 5.77 | Chr12 | 22383334-22386920 |
| OeREM45 | GWHGAOPM025523 | REM | 214 | 642 | 25.22858 | 5.57 | Chr12 | 25414715-25415643 |
| OeREM46 | GWHGAOPM025758 | REM | 260 | 783 | 29.68796 | 8.44 | Chr12 | 30197996-30198778 |
| OeREM47 | GWHGAOPM026659 | REM | 210 | 631 | 24.76664 | 5.5 | Chr13 | 923984-924829 |
| OeREM48 | GWHGAOPM027180 | REM | 152 | 458 | 18.03923 | 6.09 | Chr13 | 7936855-7937398 |
| OeREM49 | GWHGAOPM027238 | REM | 150 | 451 | 17.91225 | 6.22 | Chr13 | 8828835-8830912 |
| OeREM50 | GWHGAOPM028253 | REM | 145 | 434 | 16.73008 | 4.88 | Chr13 | 28193975-28197858 |
| OeARF25 | GWHGAOPM028413 | ARF | 657 | 1961 | 73.64032 | 6.67 | Chr13 | 32177040-32181945 |
| OeRAV11 | GWHGAOPM028473 | RAV | 405 | 1215 | 45.33398 | 6.29 | Chr13 | 33531090-33533126 |
| OeREM51 | GWHGAOPM028749 | REM | 161 | 484 | 19.0925 | 5.55 | Chr13 | 41523604-41525741 |
| OeHSI5 | GWHGAOPM029166 | HSI | 897 | 2683 | 98.61932 | 6.23 | Chr13 | 50845071-50852835 |
| OeREM52 | GWHGAOPM029226 | REM | 284 | 850 | 32.58967 | 9.62 | Chr14 | 240721-242061 |
| OeREM53 | GWHGAOPM029260 | REM | 183 | 550 | 21.50801 | 5.76 | Chr14 | 893208-893937 |
| OeARF26 | GWHGAOPM029659 | ARF | 935 | 2795 | 103.7864 | 5.4 | Chr14 | 6792788-6799916 |
| OeREM54 | GWHGAOPM030373 | REM | 237 | 712 | 27.21692 | 9.92 | Chr14 | 21160799-21161625 |
| OeLAV2 | GWHGAOPM030431 | LAV | 254 | 761 | 29.03979 | 5.44 | Chr14 | 22603059-22608346 |
| OeREM55 | GWHGAOPM030592 | REM | 133 | 400 | 15.78216 | 8.51 | Chr14 | 27057292-27057941 |
| OeARF27 | GWHGAOPM030978 | ARF | 587 | 1750 | 66.09956 | 9.2 | Chr15 | 303281-307753 |
| OeREM56 | GWHGAOPM031386 | REM | 301 | 903 | 35.0334 | 9.78 | Chr15 | 7293732-7302176 |
| OeREM57 | GWHGAOPM031387 | REM | 295 | 885 | 33.79551 | 9.44 | Chr15 | 7330848-7332862 |
| OeARF28 | GWHGAOPM031630 | ARF | 666 | 1988 | 74.66119 | 6.02 | Chr15 | 11510574-11519934 |
| OeREM58 | GWHGAOPM031695 | REM | 270 | 813 | 30.77264 | 9.55 | Chr15 | 12582639-12583451 |
| OeREM59 | GWHGAOPM031696 | REM | 272 | 819 | 31.22193 | 9.52 | Chr15 | 12596294-12597112 |
| OeREM60 | GWHGAOPM031697 | REM | 271 | 816 | 31.00962 | 9.25 | Chr15 | 12616714-12617529 |
| OeREM61 | GWHGAOPM031699 | REM | 272 | 819 | 31.26707 | 9.65 | Chr15 | 12653357-12654175 |
| OeREM62 | GWHGAOPM031700 | REM | 290 | 873 | 33.10009 | 9.55 | Chr15 | 12689389-12690261 |
| OeREM63 | GWHGAOPM031741 | REM | 150 | 451 | 17.23549 | 9.55 | Chr15 | 13539132-13540095 |
| OeREM64 | GWHGAOPM031977 | REM | 152 | 457 | 18.26934 | 5.73 | Chr15 | 20988726-20989362 |
| OeARF29 | GWHGAOPM032112 | ARF | 623 | 1860 | 70.38798 | 6.66 | Chr15 | 24550599-24556790 |
| OeREM65 | GWHGAOPM032214 | REM | 163 | 489 | 18.75204 | 9.15 | Chr15 | 26847380-26849404 |
| OeREM66 | GWHGAOPM032226 | REM | 261 | 783 | 29.79908 | 5.51 | Chr15 | 27104906-27109575 |
| OeREM67 | GWHGAOPM032228 | REM | 182 | 547 | 20.83428 | 5.68 | Chr15 | 27141947-27144381 |
| OeREM68 | GWHGAOPM032589 | REM | 277 | 828 | 31.61838 | 9.64 | Chr15 | 33253314-33256717 |
| OeREM69 | GWHGAOPM032590 | REM | 214 | 640 | 24.91198 | 5.14 | Chr15 | 33264715-33268585 |
| OeREM70 | GWHGAOPM032591 | REM | 492 | 1472 | 56.54338 | 5.99 | Chr15 | 33269415-33283912 |
| OeREM71 | GWHGAOPM032601 | REM | 270 | 806 | 30.90158 | 9.54 | Chr15 | 33407565-33410277 |
| OeREM72 | GWHGAOPM032603 | REM | 243 | 727 | 28.66039 | 5.33 | Chr15 | 33418736-33422618 |
| OeREM73 | GWHGAOPM032604 | REM | 463 | 1387 | 52.9156 | 5.6 | Chr15 | 33425099-33428319 |
| OeREM74 | GWHGAOPM033247 | REM | 301 | 899 | 34.54035 | 8.98 | Chr16 | 33007-37109 |
| OeREM75 | GWHGAOPM033248 | REM | 225 | 673 | 25.53521 | 9.3 | Chr16 | 50793-54912 |
| OeREM76 | GWHGAOPM033402 | REM | 216 | 648 | 25.27203 | 5.84 | Chr16 | 2516247-2520883 |
| OeREM77 | GWHGAOPM033413 | REM | 230 | 691 | 26.82297 | 5.72 | Chr16 | 2682179-2686307 |
| OeARF30 | GWHGAOPM033730 | ARF | 881 | 2632 | 97.31662 | 6.06 | Chr16 | 8864126-8870036 |
| OeREM78 | GWHGAOPM033946 | REM | 99 | 300 | 11.8269 | 8.87 | Chr16 | 15041638-15048688 |
| OeREM79 | GWHGAOPM034391 | REM | 269 | 806 | 30.86611 | 6.99 | Chr16 | 24654928-24657265 |
| OeREM80 | GWHGAOPM035576 | REM | 272 | 816 | 30.9399 | 9.66 | Chr17 | 5941231-5952879 |
| OeREM81 | GWHGAOPM035607 | REM | 190 | 571 | 22.38137 | 5.56 | Chr17 | 6380457-6381165 |
| OeREM82 | GWHGAOPM035626 | REM | 192 | 577 | 22.63754 | 5.41 | Chr17 | 6724383-6725223 |
| OeARF31 | GWHGAOPM035738 | ARF | 1040 | 3111 | 115.2357 | 5.88 | Chr17 | 8606169-8613595 |
| OeREM83 | GWHGAOPM035974 | REM | 166 | 499 | 19.62928 | 5.33 | Chr17 | 12157322-12159388 |
| OeARF32 | GWHGAOPM036122 | ARF | 681 | 2043 | 74.83186 | 6.16 | Chr17 | 14937366-14940106 |
| OeRAV12 | GWHGAOPM036211 | RAV | 271 | 814 | 30.94281 | 9.06 | Chr17 | 16639618-16642930 |
| OeARF33 | GWHGAOPM036308 | ARF | 1099 | 3284 | 121.8459 | 6.61 | Chr17 | 18634347-18641859 |
| OeLAV3 | GWHGAOPM036728 | LAV | 195 | 583 | 22.64999 | 9.49 | Chr17 | 29829184-29834502 |
| OeREM84 | GWHGAOPM036972 | REM | 403 | 1208 | 44.67601 | 5.61 | Chr17 | 35637752-35642666 |
| OeREM85 | GWHGAOPM036977 | REM | 199 | 598 | 23.51832 | 5.08 | Chr17 | 35730873-35734745 |
| OeARF34 | GWHGAOPM037318 | ARF | 798 | 2384 | 88.8096 | 6.33 | Chr17 | 42888569-42913459 |
| OeREM86 | GWHGAOPM037471 | REM | 460 | 1379 | 52.03496 | 8.73 | Chr18 | 3014164-3040478 |
| OeREM87 | GWHGAOPM038382 | REM | 312 | 936 | 35.84733 | 9.63 | Chr18 | 25630759-25633335 |
| OeREM88 | GWHGAOPM038385 | REM | 201 | 606 | 23.2538 | 10.17 | Chr18 | 25654462-25655067 |
| OeREM89 | GWHGAOPM038984 | REM | 216 | 648 | 25.53183 | 6.95 | Chr18 | 37529610-37530468 |
| OeREM90 | GWHGAOPM039119 | REM | 216 | 648 | 24.82066 | 5.48 | Chr18 | 39576852-39589409 |
| OeLAV4 | GWHGAOPM039592 | LAV | 247 | 739 | 28.03281 | 5.21 | Chr18 | 46291426-46296421 |
| OeREM91 | GWHGAOPM040171 | REM | 140 | 422 | 16.13912 | 4.72 | Chr19 | 9973156-9973663 |
| OeARF35 | GWHGAOPM040448 | ARF | 545 | 1637 | 60.83614 | 8.97 | Chr19 | 14598818-14601991 |
| OeARF36 | GWHGAOPM040846 | ARF | 665 | 1985 | 75.25366 | 6.57 | Chr19 | 21242805-21247666 |
| OeRAV13 | GWHGAOPM040896 | RAV | 429 | 1290 | 47.67132 | 6.76 | Chr19 | 22198625-22200652 |
| OeREM92 | GWHGAOPM041635 | REM | 199 | 597 | 22.48566 | 8.18 | Chr19 | 41861213-41864949 |
| OeREM93 | GWHGAOPM041636 | REM | 246 | 738 | 28.42655 | 8.08 | Chr19 | 41884161-41885779 |
| OeARF37 | GWHGAOPM041666 | ARF | 630 | 1881 | 70.93106 | 6.7 | Chr19 | 42325496-42329483 |
| OeARF38 | GWHGAOPM042595 | ARF | 633 | 1897 | 70.55739 | 6.01 | Chr20 | 7839531-7842203 |
| OeRAV14 | GWHGAOPM042688 | RAV | 281 | 844 | 32.09844 | 6.76 | Chr20 | 10359746-10362499 |
| OeHSI6 | GWHGAOPM043066 | HSI | 806 | 2409 | 88.23788 | 5.93 | Chr20 | 20677211-20683642 |
| OeARF39 | GWHGAOPM043409 | ARF | 613 | 1839 | 68.25624 | 7.94 | Chr20 | 27940918-27943901 |
| OeREM94 | GWHGAOPM043664 | REM | 174 | 524 | 19.84632 | 5.64 | Chr21 | 236360-236946 |
| OeREM95 | GWHGAOPM043680 | REM | 284 | 851 | 32.32234 | 9.12 | Chr21 | 495644-497982 |
| OeREM96 | GWHGAOPM043990 | REM | 159 | 477 | 18.55723 | 5.69 | Chr21 | 9826068-9828596 |
| OeARF40 | GWHGAOPM044039 | ARF | 876 | 2617 | 96.70981 | 5.98 | Chr21 | 10977828-10983771 |
| OeREM97 | GWHGAOPM044166 | REM | 198 | 595 | 23.48963 | 6.13 | Chr21 | 13391214-13391934 |
| OeREM98 | GWHGAOPM044174 | REM | 144 | 434 | 16.8171 | 5.74 | Chr21 | 13627629-13628146 |
| OeARF41 | GWHGAOPM044260 | ARF | 692 | 2069 | 76.58044 | 8.24 | Chr21 | 15476768-15481928 |
| OeARF42 | GWHGAOPM044270 | ARF | 697 | 2084 | 76.44914 | 6.89 | Chr21 | 15574659-15579419 |
| OeHSI7 | GWHGAOPM044339 | HSI | 851 | 2541 | 94.20482 | 7.45 | Chr21 | 16547700-16556665 |
| OeHSI8 | GWHGAOPM044362 | HSI | 881 | 2633 | 97.5215 | 6.54 | Chr21 | 16769779-16778742 |
| OeLAV5 | GWHGAOPM045268 | LAV | 748 | 2242 | 83.95748 | 6.02 | Chr22 | 2614804-2617607 |
| OeREM99 | GWHGAOPM045300 | REM | 178 | 534 | 20.71932 | 5.34 | Chr22 | 2952129-2954497 |
| OeHSI9 | GWHGAOPM045408 | HSI | 826 | 2469 | 91.32074 | 7.03 | Chr22 | 4359416-4370463 |
| OeARF43 | GWHGAOPM045502 | ARF | 702 | 2106 | 77.03455 | 6.88 | Chr22 | 5785783-5789906 |
| OeARF44 | GWHGAOPM045522 | ARF | 780 | 2332 | 86.60653 | 5.88 | Chr22 | 6158069-6171771 |
| OeREM100 | GWHGAOPM045725 | REM | 315 | 942 | 36.06191 | 5.99 | Chr22 | 9272029-9278584 |
| OeARF45 | GWHGAOPM046357 | ARF | 633 | 1888 | 71.37461 | 6.89 | Chr22 | 25874829-25880540 |
| OeREM101 | GWHGAOPM046579 | REM | 157 | 471 | 18.32758 | 4.97 | Chr22 | 31790306-31792778 |
| OeREM102 | GWHGAOPM047351 | REM | 181 | 544 | 21.40132 | 5.22 | Chr23 | 7942042-7944137 |
| OeREM103 | GWHGAOPM047827 | REM | 337 | 1010 | 38.20756 | 9.2 | Chr23 | 15577605-15581363 |
| OeREM104 | GWHGAOPM047828 | REM | 316 | 947 | 36.06612 | 8.73 | Chr23 | 15598655-15600279 |
| OeREM105 | GWHGAOPM047830 | REM | 369 | 1105 | 42.82637 | 9.42 | Chr23 | 15625982-15628453 |
| OeREM106 | GWHGAOPM047851 | REM | 190 | 569 | 21.81335 | 5.73 | Chr23 | 15996276-15998713 |
| OeARF46 | GWHGAOPM048048 | ARF | 877 | 2620 | 97.3372 | 5.99 | Chr23 | 20237334-20248017 |
| OeARF47 | GWHGAOPM048473 | ARF | 681 | 2030 | 76.74522 | 8.32 | Chr23 | 31385311-31395048 |
| OeREM107 | GWHGAOPM048778 | REM | 359 | 1077 | 40.88719 | 9.59 | Contig00026 | 57002-58476 |
| OeARF48 | GWHGAOPM048959 | ARF | 888 | 2654 | 98.77765 | 6.03 | Contig001253_ERROPOS2058135 | 185-13874 |
| OeREM108 | GWHGAOPM049000 | REM | 191 | 574 | 22.34394 | 5.41 | Contig001253_ERROPOS2445222 | 193965-194683 |
| OeREM109 | GWHGAOPM049255 | REM | 226 | 679 | 26.79799 | 5.72 | Contig001255_ERROPOS2905496 | 174487-175345 |
| OeREM110 | GWHGAOPM049556 | REM | 205 | 617 | 24.24811 | 5.98 | Contig001255_ERROPOS7496999 | 164227-164937 |
| OeREM111 | GWHGAOPM049749 | REM | 169 | 506 | 19.80318 | 4.43 | Contig001257 | 2045457-2049640 |
| OeREM112 | GWHGAOPM049750 | REM | 196 | 589 | 22.9992 | 5.84 | Contig001257 | 2051902-2052621 |
| OeREM113 | GWHGAOPM049755 | REM | 180 | 541 | 21.27721 | 6.33 | Contig001257 | 2203906-2204610 |
| OeREM114 | GWHGAOPM050347 | REM | 155 | 466 | 18.16088 | 9.32 | Contig001282_ERROPOS2351048 | 42860-43431 |
| OeREM115 | GWHGAOPM050502 | REM | 466 | 1396 | 52.26869 | 8.98 | Contig001289_ERROPOS1395744 | 56183-69995 |
| OeREM116 | GWHGAOPM050943 | REM | 169 | 506 | 19.80318 | 4.43 | Contig00200 | 2045457-2049640 |
| OeREM117 | GWHGAOPM050944 | REM | 196 | 589 | 22.9992 | 5.84 | Contig00200 | 2051902-2052621 |
| OeREM118 | GWHGAOPM050955 | REM | 180 | 541 | 21.27721 | 6.33 | Contig00200 | 2203906-2204610 |
| OeREM119 | GWHGAOPM052045 | REM | 147 | 442 | 17.26224 | 5.2 | Contig01215_ERROPOS3621428 | 115181-117219 |
| OeREM120 | GWHGAOPM052256 | REM | 205 | 617 | 24.24811 | 5.98 | Contig01228_ERROPOS15398520 | 14291-15001 |
| OeREM121 | GWHGAOPM052522 | REM | 226 | 679 | 26.79799 | 5.72 | Contig01228_ERROPOS20001093 | 3918-4776 |
| OeREM122 | GWHGAOPM052799 | REM | 537 | 1609 | 59.89351 | 8.82 | Contig01234_ERROPOS7053951 | 136290-148373 |
| OeARF49 | GWHGAOPM052974 | ARF | 885 | 2644 | 98.71167 | 5.89 | Contig01238_ERROPOS8044282 | 24802-38089 |
| OeREM123 | GWHGAOPM053013 | REM | 191 | 574 | 22.34394 | 5.41 | Contig01238_ERROPOS8658577 | 14906-15624 |

**Table S3. Duplicated genes of B3 superfamily identified in olive**

| **Duplication types** | **Gene Name1** | **Gene Name2** | **Gene ID1** | **Gene ID2** |
| --- | --- | --- | --- | --- |
| segmental duplication | OeRAV2 | OeRAV3 | GWHGAOPM001565 | GWHGAOPM010989 |
| segmental duplication | OeRAV2 | OeRAV4 | GWHGAOPM001565 | GWHGAOPM015562 |
| segmental duplication | OeARF1 | OeARF23 | GWHGAOPM000403 | GWHGAOPM024600 |
| segmental duplication | OeREM1 | OeREM109 | GWHGAOPM002123 | GWHGAOPM049255 |
| segmental duplication | OeREM1 | OeREM121 | GWHGAOPM002123 | GWHGAOPM052522 |
| segmental duplication | OeREM2 | OeREM11 | GWHGAOPM002258 | GWHGAOPM004396 |
| segmental duplication | OeARF2 | OeARF11 | GWHGAOPM002508 | GWHGAOPM013510 |
| segmental duplication | OeARF3 | OeARF22 | GWHGAOPM003114 | GWHGAOPM023961 |
| segmental duplication | OeREM2 | OeREM84 | GWHGAOPM002258 | GWHGAOPM036972 |
| segmental duplication | OeARF3 | OeARF33 | GWHGAOPM003114 | GWHGAOPM036308 |
| segmental duplication | OeARF2 | OeARF34 | GWHGAOPM002508 | GWHGAOPM037318 |
| segmental duplication | OeARF4 | OeARF31 | GWHGAOPM003954 | GWHGAOPM035738 |
| segmental duplication | OeREM6 | OeREM82 | GWHGAOPM003214 | GWHGAOPM035626 |
| segmental duplication | OeREM11 | OeREM16 | GWHGAOPM004396 | GWHGAOPM008419 |
| segmental duplication | OeARF5 | OeARF27 | GWHGAOPM005137 | GWHGAOPM030978 |
| segmental duplication | OeARF5 | OeARF29 | GWHGAOPM005137 | GWHGAOPM032112 |
| segmental duplication | OeREM12 | OeREM63 | GWHGAOPM005461 | GWHGAOPM031741 |
| segmental duplication | OeARF6 | OeARF28 | GWHGAOPM005523 | GWHGAOPM031630 |
| segmental duplication | OeREM13 | OeREM56 | GWHGAOPM005686 | GWHGAOPM031386 |
| segmental duplication | OeARF7 | OeARF27 | GWHGAOPM006011 | GWHGAOPM030978 |
| segmental duplication | OeREM11 | OeREM74 | GWHGAOPM004396 | GWHGAOPM033247 |
| segmental duplication | OeREM11 | OeREM84 | GWHGAOPM004396 | GWHGAOPM036972 |
| segmental duplication | OeARF9 | OeARF20 | GWHGAOPM006492 | GWHGAOPM022620 |
| segmental duplication | OeHSI1 | OeHSI4 | GWHGAOPM006368 | GWHGAOPM022959 |
| segmental duplication | OeARF10 | OeARF36 | GWHGAOPM007921 | GWHGAOPM040846 |
| segmental duplication | OeHSI1 | OeHSI6 | GWHGAOPM006368 | GWHGAOPM043066 |
| segmental duplication | OeARF8 | OeARF39 | GWHGAOPM006478 | GWHGAOPM043409 |
| segmental duplication | OeLAV1 | OeLAV5 | GWHGAOPM006235 | GWHGAOPM045268 |
| segmental duplication | OeHSI1 | OeHSI9 | GWHGAOPM006368 | GWHGAOPM045408 |
| segmental duplication | OeARF9 | OeARF44 | GWHGAOPM006492 | GWHGAOPM045522 |
| segmental duplication | OeARF10 | OeARF45 | GWHGAOPM007921 | GWHGAOPM046357 |
| segmental duplication | OeARF8 | OeARF43 | GWHGAOPM006478 | GWHGAOPM045502 |
| segmental duplication | OeREM16 | OeREM71 | GWHGAOPM008419 | GWHGAOPM032601 |
| segmental duplication | OeREM16 | OeREM74 | GWHGAOPM008419 | GWHGAOPM033247 |
| segmental duplication | OeRAV3 | OeRAV4 | GWHGAOPM010989 | GWHGAOPM015562 |
| segmental duplication | OeREM21 | OeREM91 | GWHGAOPM010206 | GWHGAOPM040171 |
| segmental duplication | OeREM20 | OeREM102 | GWHGAOPM009492 | GWHGAOPM047351 |
| segmental duplication | OeARF13 | OeARF26 | GWHGAOPM013821 | GWHGAOPM029659 |
| segmental duplication | OeARF12 | OeARF26 | GWHGAOPM013812 | GWHGAOPM029659 |
| segmental duplication | OeREM28 | OeREM71 | GWHGAOPM013665 | GWHGAOPM032601 |
| segmental duplication | OeARF11 | OeARF34 | GWHGAOPM013510 | GWHGAOPM037318 |
| segmental duplication | OeRAV5 | OeRAV9 | GWHGAOPM016934 | GWHGAOPM023375 |
| segmental duplication | OeARF16 | OeARF22 | GWHGAOPM017389 | GWHGAOPM023961 |
| segmental duplication | OeRAV7 | OeRAV10 | GWHGAOPM017295 | GWHGAOPM023559 |
| segmental duplication | OeREM33 | OeREM42 | GWHGAOPM017025 | GWHGAOPM023881 |
| segmental duplication | OeRAV7 | OeRAV8 | GWHGAOPM017295 | GWHGAOPM021666 |
| segmental duplication | OeHSI2 | OeHSI5 | GWHGAOPM015868 | GWHGAOPM029166 |
| segmental duplication | OeRAV7 | OeRAV12 | GWHGAOPM017295 | GWHGAOPM036211 |
| segmental duplication | OeARF16 | OeARF33 | GWHGAOPM017389 | GWHGAOPM036308 |
| segmental duplication | OeRAV7 | OeRAV14 | GWHGAOPM017295 | GWHGAOPM042688 |
| segmental duplication | OeREM37 | OeREM51 | GWHGAOPM019360 | GWHGAOPM028749 |
| segmental duplication | OeARF17 | OeARF47 | GWHGAOPM017781 | GWHGAOPM048473 |
| segmental duplication | OeRAV8 | OeRAV10 | GWHGAOPM021666 | GWHGAOPM023559 |
| segmental duplication | OeARF19 | OeARF20 | GWHGAOPM021778 | GWHGAOPM022620 |
| segmental duplication | OeARF20 | OeARF24 | GWHGAOPM022620 | GWHGAOPM025413 |
| segmental duplication | OeRAV10 | OeRAV12 | GWHGAOPM023559 | GWHGAOPM036211 |
| segmental duplication | OeRAV8 | OeRAV12 | GWHGAOPM021666 | GWHGAOPM036211 |
| segmental duplication | OeARF19 | OeARF32 | GWHGAOPM021778 | GWHGAOPM036122 |
| segmental duplication | OeARF20 | OeARF39 | GWHGAOPM022620 | GWHGAOPM043409 |
| segmental duplication | OeHSI4 | OeHSI6 | GWHGAOPM022959 | GWHGAOPM043066 |
| segmental duplication | OeRAV8 | OeRAV14 | GWHGAOPM021666 | GWHGAOPM042688 |
| segmental duplication | OeARF19 | OeARF38 | GWHGAOPM021778 | GWHGAOPM042595 |
| segmental duplication | OeARF19 | OeARF43 | GWHGAOPM021778 | GWHGAOPM045502 |
| segmental duplication | OeARF20 | OeARF43 | GWHGAOPM022620 | GWHGAOPM045502 |
| segmental duplication | OeARF24 | OeARF39 | GWHGAOPM025413 | GWHGAOPM043409 |
| segmental duplication | OeARF25 | OeARF36 | GWHGAOPM028413 | GWHGAOPM040846 |
| segmental duplication | OeRAV11 | OeRAV13 | GWHGAOPM028473 | GWHGAOPM040896 |
| segmental duplication | OeLAV2 | OeLAV3 | GWHGAOPM030431 | GWHGAOPM036728 |
| segmental duplication | OeLAV2 | OeLAV4 | GWHGAOPM030431 | GWHGAOPM039592 |
| segmental duplication | OeARF27 | OeARF29 | GWHGAOPM030978 | GWHGAOPM032112 |
| segmental duplication | OeREM71 | OeREM74 | GWHGAOPM032601 | GWHGAOPM033247 |
| segmental duplication | OeREM64 | OeREM111 | GWHGAOPM031977 | GWHGAOPM049749 |
| segmental duplication | OeREM64 | OeREM116 | GWHGAOPM031977 | GWHGAOPM050943 |
| segmental duplication | OeARF30 | OeARF40 | GWHGAOPM033730 | GWHGAOPM044039 |
| segmental duplication | OeLAV3 | OeLAV4 | GWHGAOPM036728 | GWHGAOPM039592 |
| segmental duplication | OeARF32 | OeARF38 | GWHGAOPM036122 | GWHGAOPM042595 |
| segmental duplication | OeRAV12 | OeRAV14 | GWHGAOPM036211 | GWHGAOPM042688 |
| segmental duplication | OeARF35 | OeARF38 | GWHGAOPM040448 | GWHGAOPM042595 |
| segmental duplication | OeARF36 | OeARF45 | GWHGAOPM040846 | GWHGAOPM046357 |
| segmental duplication | OeHSI6 | OeHSI9 | GWHGAOPM043066 | GWHGAOPM045408 |
| segmental duplication | OeARF39 | OeARF43 | GWHGAOPM043409 | GWHGAOPM045502 |
| segmental duplication | OeARF48 | OeARF49 | GWHGAOPM048959 | GWHGAOPM052974 |
| segmental duplication | OeREM109 | OeREM121 | GWHGAOPM049255 | GWHGAOPM052522 |
| segmental duplication | OeREM110 | OeREM120 | GWHGAOPM049556 | GWHGAOPM052256 |
| segmental duplication | OeREM111 | OeREM116 | GWHGAOPM049749 | GWHGAOPM050943 |
| segmental duplication | OeREM112 | OeREM117 | GWHGAOPM049750 | GWHGAOPM050944 |
| segmental duplication | OeREM113 | OeREM118 | GWHGAOPM049755 | GWHGAOPM050955 |
| tandem duplication | OeARF13 | OeARF14 | GWHGAOPM013821 | GWHGAOPM013822 |
| tandem duplication | OeREM56 | OeREM57 | GWHGAOPM031386 | GWHGAOPM031387 |
| tandem duplication | OeREM58 | OeREM59 | GWHGAOPM031695 | GWHGAOPM031696 |
| tandem duplication | OeREM59 | OeREM60 | GWHGAOPM031696 | GWHGAOPM031697 |
| tandem duplication | OeREM61 | OeREM62 | GWHGAOPM031699 | GWHGAOPM031700 |
| tandem duplication | OeREM69 | OeREM70 | GWHGAOPM032590 | GWHGAOPM032591 |
| tandem duplication | OeREM72 | OeREM73 | GWHGAOPM032603 | GWHGAOPM032604 |
| tandem duplication | OeREM74 | OeREM75 | GWHGAOPM033247 | GWHGAOPM033248 |
| tandem duplication | OeREM92 | OeREM93 | GWHGAOPM041635 | GWHGAOPM041636 |
| tandem duplication | OeREM103 | OeREM104 | GWHGAOPM047827 | GWHGAOPM047828 |

**Table S4. The duplicated genes of B3 superfamily between olive and Arabidopsis and between olive and rice**

| **Gene Name1** | **Gene Name2** |
| --- | --- |
| OeRAV3 | AT1G13260 |
| OeRAV4 | AT1G13260 |
| OeARF12 | AT1G19850 |
| OeARF13 | AT1G19850 |
| OeARF26 | AT1G19850 |
| OeRAV2 | AT1G25560 |
| OeRAV3 | AT1G25560 |
| OeRAV4 | AT1G25560 |
| OeARF30 | AT1G30330 |
| OeARF40 | AT1G30330 |
| OeREM57 | AT1G49475 |
| OeRAV3 | AT1G68840 |
| OeRAV4 | AT1G68840 |
| OeARF15 | AT1G77850 |
| OeARF18 | AT1G77850 |
| OeARF18 | AT1G77850 |
| OeARF8 | AT2G28350 |
| OeARF20 | AT2G28350 |
| OeARF19 | AT2G28350 |
| OeARF24 | AT2G28350 |
| OeARF39 | AT2G28350 |
| OeARF43 | AT2G28350 |
| OeHSI1 | AT2G30470 |
| OeHSI6 | AT2G30470 |
| OeHSI9 | AT2G30470 |
| OeARF41 | AT2G33860 |
| OeRAV7 | AT2G36080 |
| OeRAV8 | AT2G36080 |
| OeRAV10 | AT2G36080 |
| OeRAV12 | AT2G36080 |
| OeRAV14 | AT2G36080 |
| OeARF7 | AT2G46530 |
| OeARF5 | AT2G46530 |
| OeARF25 | AT2G46530 |
| OeARF29 | AT2G46530 |
| OeARF27 | AT2G46530 |
| OeARF36 | AT2G46530 |
| OeARF45 | AT2G46530 |
| OeRAV13 | AT2G46870 |
| OeRAV7 | AT3G11580 |
| OeRAV10 | AT3G11580 |
| OeRAV12 | AT3G11580 |
| OeREM13 | AT3G18960 |
| OeREM11 | AT3G19184 |
| OeREM70 | AT3G19184 |
| OeREM75 | AT3G19184 |
| OeLAV1 | AT3G24650 |
| OeLAV5 | AT3G24650 |
| OeRAV2 | AT3G25730 |
| OeRAV3 | AT3G25730 |
| OeRAV4 | AT3G25730 |
| OeLAV3 | AT3G26790 |
| OeLAV4 | AT3G26790 |
| OeARF7 | AT3G61830 |
| OeARF5 | AT3G61830 |
| OeARF29 | AT3G61830 |
| OeHSI3 | AT4G21550 |
| OeARF10 | AT4G23980 |
| OeARF25 | AT4G23980 |
| OeARF27 | AT4G23980 |
| OeARF36 | AT4G23980 |
| OeARF45 | AT4G23980 |
| OeRAV7 | AT5G06250 |
| OeRAV10 | AT5G06250 |
| OeARF31 | AT5G20730 |
| OeREM14 | AT5G58280 |
| OeARF9 | AT5G60450 |
| OeARF44 | AT5G60450 |
| OeREM14 | Os05g40280 |
| OeARF42 | Os05g48870 |
| OeHSI8 | Os07g37610 |
| OeHSI1 | Os07g48200 |
| OeHSI9 | Os07g48200 |
| OeARF8 | Os10g33940 |
| OeRAV7 | Os11g05740 |
| OeRAV10 | Os11g05740 |
| OeRAV12 | Os11g05740 |
| OeRAV7 | Os12g06080 |
| OeRAV12 | Os12g06080 |

**Table S5. The largest syntenic blocks between olive and Arabidopsis and between olive and rice**

| #1 . The largest syntenic blocks between olive and Arabidopsis | | |  |
| --- | --- | --- | --- |
| **ID in Block** | **Gene in Olive** | **Gene in Arabidopsis** | **Pvalue** |
| 1 | GWHGAOPM021079 | AT5G53810 | 1.00E-82 |
| 2 | GWHGAOPM021080 | AT5G53730 | 2.00E-25 |
| 3 | GWHGAOPM021091 | AT5G53660 | 2.00E-34 |
| 4 | GWHGAOPM021092 | AT5G53620 | 3.00E-66 |
| 5 | GWHGAOPM021103 | AT5G53570 | 1.00E-160 |
| 6 | GWHGAOPM021104 | AT5G53560 | 3.00E-47 |
| 7 | GWHGAOPM021105 | AT5G53550 | 3.00E-276 |
| 8 | GWHGAOPM021106 | AT5G53540 | 3.00E-89 |
| 9 | GWHGAOPM021107 | AT5G53530 | 1.00E-137 |
| 10 | GWHGAOPM021114 | AT5G53500 | 9.00E-130 |
| 11 | GWHGAOPM021118 | AT5G53480 | 0 |
| 12 | GWHGAOPM021136 | AT5G53350 | 2.00E-189 |
| 13 | GWHGAOPM021152 | AT5G53150 | 6.00E-138 |
| 14 | GWHGAOPM021166 | AT5G52950 | 1.00E-99 |
| 15 | GWHGAOPM021175 | AT5G52920 | 1.00E-258 |
| 16 | GWHGAOPM021176 | AT5G52910 | 5.00E-270 |
| 17 | GWHGAOPM021178 | AT5G52900 | 1.00E-26 |
| 18 | GWHGAOPM021181 | AT5G52882 | 3.00E-294 |
| 19 | GWHGAOPM021187 | AT5G52860 | 8.00E-218 |
| 20 | GWHGAOPM021191 | AT5G52840 | 4.00E-66 |
| 21 | GWHGAOPM021192 | AT5G52830 | 1.00E-30 |
| 22 | GWHGAOPM021194 | AT5G52800 | 3.00E-142 |
| 23 | GWHGAOPM021198 | AT5G52740 | 7.00E-15 |
| 24 | GWHGAOPM021202 | AT5G52650 | 3.00E-44 |
| 25 | GWHGAOPM021205 | AT5G52540 | 2.00E-155 |
| 26 | GWHGAOPM021207 | AT5G52520 | 3.00E-257 |
| 27 | GWHGAOPM021216 | AT5G52430 | 2.00E-91 |
| 28 | GWHGAOPM021228 | AT5G52410 | 8.00E-102 |
| 29 | GWHGAOPM021231 | AT5G52400 | 1.00E-193 |
| 30 | GWHGAOPM021232 | AT5G52380 | 5.00E-11 |
| 31 | GWHGAOPM021233 | AT5G52360 | 2.00E-63 |
| 32 | GWHGAOPM021238 | AT5G52300 | 9.00E-37 |
| 33 | GWHGAOPM021242 | AT5G52260 | 4.00E-50 |
| 34 | GWHGAOPM021245 | AT5G52240 | 3.00E-65 |
| 35 | GWHGAOPM021248 | AT5G52210 | 3.00E-82 |
| 36 | GWHGAOPM021260 | AT5G52120 | 4.00E-80 |
| 37 | GWHGAOPM021262 | AT5G52110 | 2.00E-60 |
| 38 | GWHGAOPM021263 | AT5G52060 | 7.00E-71 |
| 39 | GWHGAOPM021264 | AT5G52050 | 1.00E-131 |
| 40 | GWHGAOPM021265 | AT5G52040 | 2.00E-73 |
| 41 | GWHGAOPM021268 | AT5G51990 | 7.00E-32 |
| 42 | GWHGAOPM021272 | AT5G51980 | 1.00E-128 |
| 43 | GWHGAOPM021273 | AT5G51970 | 2.00E-177 |
| 44 | GWHGAOPM021275 | AT5G51920 | 1.00E-149 |
| 45 | GWHGAOPM021276 | AT5G51890 | 3.00E-129 |
| 46 | GWHGAOPM021277 | AT5G51850 | 6.00E-43 |
| 47 | GWHGAOPM021278 | AT5G51840 | 9.00E-59 |
| 48 | GWHGAOPM021279 | AT5G51820 | 1.00E-293 |
| 49 | GWHGAOPM021280 | AT5G51810 | 9.00E-138 |
| 50 | GWHGAOPM021281 | AT5G51800 | 9.00E-259 |
| 51 | GWHGAOPM021282 | AT5G51780 | 5.00E-27 |
| 52 | GWHGAOPM021285 | AT5G51770 | 6.00E-106 |
| 53 | GWHGAOPM021290 | AT5G51760 | 8.00E-71 |
| 54 | GWHGAOPM021291 | AT5G51750 | 0 |
| 55 | GWHGAOPM021295 | AT5G51720 | 1.00E-31 |
| 56 | GWHGAOPM021296 | AT5G51710 | 8.00E-245 |
| 57 | GWHGAOPM021298 | AT5G51670 | 3.00E-110 |
| 58 | GWHGAOPM021299 | AT5G51640 | 2.00E-173 |
| 59 | GWHGAOPM021304 | AT5G51570 | 8.00E-142 |
| 60 | GWHGAOPM021308 | AT5G51550 | 5.00E-154 |
| 61 | GWHGAOPM021311 | AT5G51520 | 4.00E-18 |
| 62 | GWHGAOPM021314 | AT5G51490 | 6.00E-160 |
| 63 | GWHGAOPM021319 | AT5G51460 | 6.00E-140 |
| 64 | GWHGAOPM021324 | AT5G51450 | 1.00E-176 |
|  |  |  |  |
|  |  |  |  |
| #2 . The largest syntenic blocks between olive and rice | | |  |
| **ID in Block** | **Gene in Olive** | **Gene in rice** | **Pvalue** |
| 1 | GWHGAOPM050790 | Os10g21192 | 5.00E-203 |
| 2 | GWHGAOPM050789 | Os10g21194 | 5.00E-143 |
| 3 | GWHGAOPM050788 | Os10g21196 | 4.00E-18 |
| 4 | GWHGAOPM050787 | Os10g21198 | 1.00E-20 |
| 5 | GWHGAOPM050778 | Os10g21212 | 6.00E-277 |
| 6 | GWHGAOPM050777 | Os10g21214 | 1.00E-22 |
| 7 | GWHGAOPM050776 | Os10g21244 | 1.00E-30 |
| 8 | GWHGAOPM050774 | Os10g21248 | 2.00E-144 |
| 9 | GWHGAOPM050773 | Os10g21254 | 5.00E-65 |
| 10 | GWHGAOPM050771 | Os10g21258 | 7.00E-82 |
| 11 | GWHGAOPM050768 | Os10g21266 | 2.00E-239 |
| 12 | GWHGAOPM050767 | Os10g21268 | 3.00E-272 |
| 13 | GWHGAOPM050763 | Os10g21290 | 1.00E-169 |
| 14 | GWHGAOPM050762 | Os10g21298 | 8.00E-42 |
| 15 | GWHGAOPM050761 | Os10g21308 | 5.00E-23 |
| 16 | GWHGAOPM050760 | Os10g21312 | 5.00E-24 |
| 17 | GWHGAOPM050759 | Os10g21314 | 2.00E-35 |
| 18 | GWHGAOPM050758 | Os10g21300 | 5.00E-77 |
| 19 | GWHGAOPM050757 | Os10g21310 | 8.00E-302 |
| 20 | GWHGAOPM050755 | Os10g21322 | 5.00E-30 |
| 21 | GWHGAOPM050754 | Os10g21324 | 3.00E-120 |
| 22 | GWHGAOPM050753 | Os10g21326 | 5.00E-76 |
| 23 | GWHGAOPM050752 | Os10g21328 | 8.00E-130 |
| 24 | GWHGAOPM050751 | Os10g21332 | 9.00E-46 |
| 25 | GWHGAOPM050749 | Os10g21342 | 4.00E-52 |
| 26 | GWHGAOPM050748 | Os10g21344 | 5.00E-56 |
| 27 | GWHGAOPM050747 | Os10g21346 | 5.00E-70 |
| 28 | GWHGAOPM050745 | Os10g21358 | 1.00E-43 |
| 29 | GWHGAOPM050742 | Os10g21372 | 1.00E-29 |

**Table S6. Hormone-related cis-elements identified in the B3 superfamily in olive**

| **Gene Name** | **Gene ID** | **Gibberellin responsiveness element** | **MeJA responsiveness element** | **Auxin responsiveness element** | **Abscisic acid responsiveness element** | **Salicylic acid responsiveness element** |
| --- | --- | --- | --- | --- | --- | --- |
| OeARF1 | GWHGAOPM000403 | 0 | 0 | 0 | 1 | 0 |
| OeARF10 | GWHGAOPM007921 | 1 | 6 | 1 | 5 | 1 |
| OeARF11 | GWHGAOPM013510 | 0 | 2 | 1 | 1 | 1 |
| OeARF12 | GWHGAOPM013812 | 1 | 0 | 3 | 9 | 1 |
| OeARF13 | GWHGAOPM013821 | 1 | 0 | 2 | 3 | 1 |
| OeARF14 | GWHGAOPM013822 | 0 | 0 | 3 | 9 | 1 |
| OeARF15 | GWHGAOPM014242 | 0 | 8 | 0 | 2 | 0 |
| OeARF16 | GWHGAOPM017389 | 1 | 4 | 1 | 1 | 1 |
| OeARF17 | GWHGAOPM017781 | 3 | 10 | 1 | 3 | 0 |
| OeARF18 | GWHGAOPM018885 | 0 | 6 | 0 | 5 | 0 |
| OeARF19 | GWHGAOPM021778 | 1 | 0 | 0 | 0 | 0 |
| OeARF2 | GWHGAOPM002508 | 0 | 0 | 1 | 2 | 1 |
| OeARF20 | GWHGAOPM022620 | 1 | 2 | 2 | 2 | 1 |
| OeARF21 | GWHGAOPM022731 | 3 | 4 | 0 | 3 | 1 |
| OeARF22 | GWHGAOPM023961 | 1 | 2 | 0 | 0 | 0 |
| OeARF23 | GWHGAOPM024600 | 1 | 6 | 0 | 2 | 0 |
| OeARF24 | GWHGAOPM025413 | 0 | 4 | 1 | 1 | 0 |
| OeARF25 | GWHGAOPM028413 | 0 | 4 | 2 | 1 | 0 |
| OeARF26 | GWHGAOPM029659 | 0 | 6 | 2 | 3 | 0 |
| OeARF27 | GWHGAOPM030978 | 1 | 2 | 1 | 7 | 0 |
| OeARF28 | GWHGAOPM031630 | 1 | 2 | 1 | 2 | 0 |
| OeARF29 | GWHGAOPM032112 | 1 | 2 | 1 | 1 | 0 |
| OeARF3 | GWHGAOPM003114 | 1 | 0 | 0 | 1 | 0 |
| OeARF30 | GWHGAOPM033730 | 2 | 0 | 1 | 0 | 0 |
| OeARF31 | GWHGAOPM035738 | 0 | 2 | 1 | 1 | 1 |
| OeARF32 | GWHGAOPM036122 | 1 | 2 | 0 | 2 | 0 |
| OeARF33 | GWHGAOPM036308 | 2 | 6 | 0 | 1 | 1 |
| OeARF34 | GWHGAOPM037318 | 0 | 0 | 1 | 1 | 1 |
| OeARF35 | GWHGAOPM040448 | 0 | 4 | 1 | 2 | 2 |
| OeARF36 | GWHGAOPM040846 | 2 | 6 | 0 | 11 | 2 |
| OeARF37 | GWHGAOPM041666 | 2 | 8 | 0 | 2 | 0 |
| OeARF38 | GWHGAOPM042595 | 2 | 0 | 1 | 4 | 1 |
| OeARF39 | GWHGAOPM043409 | 0 | 6 | 1 | 0 | 0 |
| OeARF4 | GWHGAOPM003954 | 1 | 4 | 0 | 1 | 0 |
| OeARF40 | GWHGAOPM044039 | 0 | 2 | 0 | 3 | 0 |
| OeARF41 | GWHGAOPM044260 | 3 | 2 | 1 | 3 | 0 |
| OeARF42 | GWHGAOPM044270 | 4 | 0 | 1 | 3 | 1 |
| OeARF43 | GWHGAOPM045502 | 0 | 2 | 0 | 1 | 1 |
| OeARF44 | GWHGAOPM045522 | 1 | 2 | 0 | 2 | 1 |
| OeARF45 | GWHGAOPM046357 | 0 | 0 | 1 | 1 | 1 |
| OeARF46 | GWHGAOPM048048 | 0 | 4 | 0 | 1 | 2 |
| OeARF47 | GWHGAOPM048473 | 1 | 2 | 1 | 2 | 4 |
| OeARF48 | GWHGAOPM048959 | 2 | 2 | 2 | 2 | 0 |
| OeARF49 | GWHGAOPM052974 | 2 | 2 | 2 | 2 | 0 |
| OeARF5 | GWHGAOPM005137 | 2 | 4 | 1 | 1 | 1 |
| OeARF6 | GWHGAOPM005523 | 0 | 4 | 0 | 1 | 2 |
| OeARF7 | GWHGAOPM006011 | 1 | 0 | 2 | 6 | 1 |
| OeARF8 | GWHGAOPM006478 | 3 | 0 | 1 | 0 | 0 |
| OeARF9 | GWHGAOPM006492 | 2 | 4 | 0 | 2 | 1 |
| OeHSI1 | GWHGAOPM006368 | 1 | 4 | 0 | 0 | 0 |
| OeHSI2 | GWHGAOPM015868 | 2 | 0 | 0 | 2 | 1 |
| OeHSI3 | GWHGAOPM019835 | 1 | 10 | 2 | 2 | 0 |
| OeHSI4 | GWHGAOPM022959 | 0 | 0 | 0 | 7 | 1 |
| OeHSI5 | GWHGAOPM029166 | 2 | 4 | 0 | 2 | 0 |
| OeHSI6 | GWHGAOPM043066 | 2 | 0 | 0 | 2 | 0 |
| OeHSI7 | GWHGAOPM044339 | 0 | 6 | 0 | 2 | 0 |
| OeHSI8 | GWHGAOPM044362 | 0 | 6 | 0 | 2 | 0 |
| OeHSI9 | GWHGAOPM045408 | 0 | 2 | 0 | 0 | 0 |
| OeLAV1 | GWHGAOPM006235 | 1 | 0 | 1 | 0 | 0 |
| OeLAV2 | GWHGAOPM030431 | 1 | 6 | 1 | 2 | 1 |
| OeLAV3 | GWHGAOPM036728 | 1 | 0 | 0 | 2 | 1 |
| OeLAV4 | GWHGAOPM039592 | 0 | 2 | 1 | 0 | 1 |
| OeLAV5 | GWHGAOPM045268 | 0 | 6 | 1 | 4 | 0 |
| OeRAV1 | GWHGAOPM000109 | 2 | 0 | 1 | 2 | 0 |
| OeRAV10 | GWHGAOPM023559 | 1 | 4 | 0 | 2 | 2 |
| OeRAV11 | GWHGAOPM028473 | 0 | 0 | 1 | 1 | 0 |
| OeRAV12 | GWHGAOPM036211 | 2 | 0 | 1 | 0 | 0 |
| OeRAV13 | GWHGAOPM040896 | 0 | 4 | 0 | 2 | 1 |
| OeRAV14 | GWHGAOPM042688 | 0 | 2 | 0 | 1 | 2 |
| OeRAV2 | GWHGAOPM001565 | 0 | 6 | 0 | 8 | 2 |
| OeRAV3 | GWHGAOPM010989 | 0 | 4 | 0 | 0 | 1 |
| OeRAV4 | GWHGAOPM015562 | 1 | 0 | 0 | 0 | 0 |
| OeRAV5 | GWHGAOPM016934 | 0 | 0 | 0 | 4 | 0 |
| OeRAV6 | GWHGAOPM016936 | 2 | 2 | 0 | 2 | 0 |
| OeRAV7 | GWHGAOPM017295 | 1 | 2 | 1 | 5 | 1 |
| OeRAV8 | GWHGAOPM021666 | 2 | 0 | 0 | 5 | 1 |
| OeRAV9 | GWHGAOPM023375 | 2 | 4 | 1 | 3 | 0 |
| OeREM1 | GWHGAOPM002123 | 1 | 2 | 1 | 1 | 0 |
| OeREM10 | GWHGAOPM003492 | 0 | 4 | 1 | 4 | 2 |
| OeREM100 | GWHGAOPM045725 | 1 | 2 | 0 | 7 | 2 |
| OeREM101 | GWHGAOPM046579 | 0 | 2 | 1 | 2 | 1 |
| OeREM102 | GWHGAOPM047351 | 0 | 2 | 0 | 0 | 2 |
| OeREM103 | GWHGAOPM047827 | 0 | 2 | 2 | 5 | 0 |
| OeREM104 | GWHGAOPM047828 | 0 | 2 | 0 | 3 | 1 |
| OeREM105 | GWHGAOPM047830 | 3 | 2 | 2 | 2 | 1 |
| OeREM106 | GWHGAOPM047851 | 0 | 0 | 1 | 3 | 3 |
| OeREM107 | GWHGAOPM048778 | 1 | 0 | 0 | 5 | 0 |
| OeREM108 | GWHGAOPM049000 | 3 | 0 | 2 | 4 | 2 |
| OeREM109 | GWHGAOPM049255 | 1 | 2 | 1 | 1 | 0 |
| OeREM11 | GWHGAOPM004396 | 1 | 4 | 0 | 1 | 1 |
| OeREM110 | GWHGAOPM049556 | 1 | 0 | 1 | 1 | 1 |
| OeREM111 | GWHGAOPM049749 | 0 | 2 | 0 | 1 | 0 |
| OeREM112 | GWHGAOPM049750 | 0 | 0 | 2 | 3 | 1 |
| OeREM113 | GWHGAOPM049755 | 0 | 4 | 0 | 2 | 0 |
| OeREM114 | GWHGAOPM050347 | 2 | 4 | 0 | 2 | 2 |
| OeREM115 | GWHGAOPM050502 | 2 | 2 | 1 | 4 | 0 |
| OeREM116 | GWHGAOPM050943 | 0 | 2 | 0 | 1 | 0 |
| OeREM117 | GWHGAOPM050944 | 0 | 0 | 2 | 3 | 1 |
| OeREM118 | GWHGAOPM050955 | 0 | 4 | 0 | 2 | 0 |
| OeREM119 | GWHGAOPM052045 | 0 | 0 | 0 | 0 | 0 |
| OeREM12 | GWHGAOPM005461 | 0 | 6 | 0 | 0 | 0 |
| OeREM120 | GWHGAOPM052256 | 1 | 0 | 1 | 1 | 1 |
| OeREM121 | GWHGAOPM052522 | 1 | 2 | 1 | 1 | 0 |
| OeREM122 | GWHGAOPM052799 | 0 | 2 | 0 | 3 | 0 |
| OeREM123 | GWHGAOPM053013 | 3 | 0 | 2 | 4 | 2 |
| OeREM13 | GWHGAOPM005686 | 0 | 6 | 1 | 1 | 1 |
| OeREM14 | GWHGAOPM006356 | 0 | 2 | 0 | 5 | 0 |
| OeREM15 | GWHGAOPM006494 | 1 | 0 | 0 | 1 | 0 |
| OeREM16 | GWHGAOPM008419 | 0 | 0 | 1 | 1 | 0 |
| OeREM17 | GWHGAOPM008667 | 0 | 2 | 1 | 3 | 0 |
| OeREM18 | GWHGAOPM008736 | 0 | 2 | 0 | 5 | 0 |
| OeREM19 | GWHGAOPM009450 | 1 | 0 | 2 | 4 | 0 |
| OeREM2 | GWHGAOPM002258 | 2 | 4 | 1 | 3 | 1 |
| OeREM20 | GWHGAOPM009492 | 0 | 0 | 1 | 0 | 1 |
| OeREM21 | GWHGAOPM010206 | 1 | 0 | 0 | 2 | 1 |
| OeREM22 | GWHGAOPM010882 | 1 | 2 | 0 | 1 | 0 |
| OeREM23 | GWHGAOPM011965 | 3 | 0 | 2 | 5 | 0 |
| OeREM24 | GWHGAOPM012017 | 0 | 2 | 0 | 1 | 0 |
| OeREM25 | GWHGAOPM012711 | 0 | 0 | 2 | 6 | 2 |
| OeREM26 | GWHGAOPM013363 | 0 | 2 | 1 | 4 | 1 |
| OeREM27 | GWHGAOPM013568 | 0 | 0 | 0 | 1 | 0 |
| OeREM28 | GWHGAOPM013665 | 0 | 0 | 1 | 1 | 0 |
| OeREM29 | GWHGAOPM013703 | 0 | 6 | 0 | 3 | 0 |
| OeREM3 | GWHGAOPM002359 | 1 | 0 | 0 | 1 | 0 |
| OeREM30 | GWHGAOPM014233 | 0 | 0 | 2 | 4 | 0 |
| OeREM31 | GWHGAOPM015141 | 1 | 0 | 1 | 3 | 0 |
| OeREM32 | GWHGAOPM015541 | 1 | 2 | 0 | 2 | 1 |
| OeREM33 | GWHGAOPM017025 | 0 | 0 | 0 | 5 | 0 |
| OeREM34 | GWHGAOPM017983 | 2 | 6 | 0 | 2 | 0 |
| OeREM35 | GWHGAOPM018456 | 0 | 2 | 1 | 4 | 1 |
| OeREM36 | GWHGAOPM019173 | 2 | 0 | 2 | 4 | 2 |
| OeREM37 | GWHGAOPM019360 | 0 | 2 | 0 | 4 | 0 |
| OeREM38 | GWHGAOPM020311 | 2 | 2 | 0 | 0 | 2 |
| OeREM39 | GWHGAOPM021658 | 1 | 4 | 1 | 2 | 0 |
| OeREM4 | GWHGAOPM002925 | 1 | 0 | 0 | 2 | 0 |
| OeREM40 | GWHGAOPM022677 | 0 | 0 | 2 | 5 | 1 |
| OeREM41 | GWHGAOPM022678 | 0 | 0 | 0 | 2 | 0 |
| OeREM42 | GWHGAOPM023881 | 0 | 2 | 1 | 2 | 0 |
| OeREM43 | GWHGAOPM024520 | 3 | 2 | 0 | 3 | 2 |
| OeREM44 | GWHGAOPM024977 | 1 | 2 | 1 | 4 | 0 |
| OeREM45 | GWHGAOPM025523 | 0 | 0 | 0 | 3 | 1 |
| OeREM46 | GWHGAOPM025758 | 2 | 2 | 0 | 1 | 0 |
| OeREM47 | GWHGAOPM026659 | 3 | 0 | 2 | 3 | 2 |
| OeREM48 | GWHGAOPM027180 | 4 | 0 | 1 | 4 | 2 |
| OeREM49 | GWHGAOPM027238 | 1 | 2 | 1 | 2 | 0 |
| OeREM5 | GWHGAOPM003018 | 2 | 0 | 1 | 0 | 1 |
| OeREM50 | GWHGAOPM028253 | 1 | 2 | 0 | 1 | 1 |
| OeREM51 | GWHGAOPM028749 | 1 | 0 | 0 | 1 | 0 |
| OeREM52 | GWHGAOPM029226 | 3 | 4 | 1 | 4 | 0 |
| OeREM53 | GWHGAOPM029260 | 1 | 2 | 0 | 4 | 0 |
| OeREM54 | GWHGAOPM030373 | 1 | 0 | 1 | 5 | 0 |
| OeREM55 | GWHGAOPM030592 | 0 | 2 | 1 | 3 | 1 |
| OeREM56 | GWHGAOPM031386 | 1 | 2 | 1 | 3 | 3 |
| OeREM57 | GWHGAOPM031387 | 2 | 2 | 0 | 4 | 0 |
| OeREM58 | GWHGAOPM031695 | 1 | 2 | 0 | 2 | 0 |
| OeREM59 | GWHGAOPM031696 | 1 | 2 | 0 | 0 | 1 |
| OeREM6 | GWHGAOPM003214 | 4 | 0 | 1 | 3 | 1 |
| OeREM60 | GWHGAOPM031697 | 0 | 2 | 0 | 1 | 0 |
| OeREM61 | GWHGAOPM031699 | 0 | 4 | 0 | 2 | 0 |
| OeREM62 | GWHGAOPM031700 | 1 | 4 | 0 | 2 | 1 |
| OeREM63 | GWHGAOPM031741 | 2 | 16 | 2 | 8 | 0 |
| OeREM64 | GWHGAOPM031977 | 3 | 0 | 0 | 2 | 1 |
| OeREM65 | GWHGAOPM032214 | 0 | 2 | 1 | 2 | 1 |
| OeREM66 | GWHGAOPM032226 | 0 | 2 | 0 | 4 | 0 |
| OeREM67 | GWHGAOPM032228 | 2 | 6 | 1 | 3 | 0 |
| OeREM68 | GWHGAOPM032589 | 1 | 2 | 0 | 0 | 1 |
| OeREM69 | GWHGAOPM032590 | 0 | 6 | 0 | 0 | 2 |
| OeREM7 | GWHGAOPM003221 | 2 | 0 | 0 | 6 | 1 |
| OeREM70 | GWHGAOPM032591 | 0 | 2 | 1 | 2 | 3 |
| OeREM71 | GWHGAOPM032601 | 0 | 0 | 0 | 2 | 2 |
| OeREM72 | GWHGAOPM032603 | 0 | 6 | 0 | 1 | 2 |
| OeREM73 | GWHGAOPM032604 | 2 | 0 | 0 | 3 | 1 |
| OeREM74 | GWHGAOPM033247 | 1 | 4 | 0 | 2 | 0 |
| OeREM75 | GWHGAOPM033248 | 5 | 6 | 1 | 1 | 1 |
| OeREM76 | GWHGAOPM033402 | 0 | 2 | 0 | 0 | 1 |
| OeREM77 | GWHGAOPM033413 | 0 | 0 | 0 | 0 | 1 |
| OeREM78 | GWHGAOPM033946 | 0 | 2 | 0 | 5 | 2 |
| OeREM79 | GWHGAOPM034391 | 2 | 0 | 0 | 0 | 0 |
| OeREM8 | GWHGAOPM003280 | 0 | 2 | 3 | 0 | 1 |
| OeREM80 | GWHGAOPM035576 | 1 | 2 | 0 | 1 | 1 |
| OeREM81 | GWHGAOPM035607 | 1 | 0 | 1 | 4 | 2 |
| OeREM82 | GWHGAOPM035626 | 1 | 2 | 0 | 1 | 0 |
| OeREM83 | GWHGAOPM035974 | 0 | 0 | 1 | 1 | 1 |
| OeREM84 | GWHGAOPM036972 | 1 | 2 | 0 | 2 | 0 |
| OeREM85 | GWHGAOPM036977 | 0 | 2 | 0 | 0 | 0 |
| OeREM86 | GWHGAOPM037471 | 1 | 2 | 0 | 1 | 0 |
| OeREM87 | GWHGAOPM038382 | 0 | 6 | 0 | 2 | 1 |
| OeREM88 | GWHGAOPM038385 | 0 | 8 | 1 | 9 | 0 |
| OeREM89 | GWHGAOPM038984 | 4 | 0 | 1 | 4 | 1 |
| OeREM9 | GWHGAOPM003382 | 0 | 0 | 0 | 2 | 0 |
| OeREM90 | GWHGAOPM039119 | 0 | 0 | 0 | 1 | 0 |
| OeREM91 | GWHGAOPM040171 | 1 | 0 | 0 | 5 | 1 |
| OeREM92 | GWHGAOPM041635 | 0 | 2 | 3 | 4 | 0 |
| OeREM93 | GWHGAOPM041636 | 1 | 2 | 0 | 0 | 0 |
| OeREM94 | GWHGAOPM043664 | 2 | 2 | 0 | 4 | 1 |
| OeREM95 | GWHGAOPM043680 | 1 | 0 | 1 | 3 | 0 |
| OeREM96 | GWHGAOPM043990 | 0 | 2 | 0 | 0 | 1 |
| OeREM97 | GWHGAOPM044166 | 2 | 2 | 2 | 1 | 1 |
| OeREM98 | GWHGAOPM044174 | 2 | 2 | 1 | 1 | 1 |
| OeREM99 | GWHGAOPM045300 | 0 | 0 | 0 | 4 | 2 |

**Table S7. The expression levels of duplicated genes of B3 superfamily in olive**

| **Gene Pairs** | **Gene1** | **Gene2** | **Gene 1** | | | | | **Gene2** | | | | |
| --- | --- | --- | --- | --- | --- | --- | --- | --- | --- | --- | --- | --- |
|  |  |  | **Fruit** | **New leaf** | **Old leaf** | **Pedicel** | **Stem** | **Fruit** | **New leaf** | **Old leaf** | **Pedicel** | **Stem** |
| OeARF1-OeARF23 | OeARF1 | OeARF23 | 26.1690 | 13.0327 | 17.0727 | 35.7170 | 35.7700 | 26.3543 | 17.4240 | 27.3950 | 25.4180 | 33.5120 |
| OeRAV2-OeRAV3 | OeRAV2 | OeRAV3 | 0.0203 | 0.3037 | 0.5640 | 4.7290 | 0.9150 | 0.5800 | 11.8660 | 105.6843 | 46.8250 | 30.6950 |
| OeRAV2-OeRAV4 | OeRAV2 | OeRAV4 | 0.0203 | 0.3037 | 0.5640 | 4.7290 | 0.9150 | 0.2657 | 2.0250 | 2.9537 | 15.3620 | 6.9520 |
| OeREM1-OeREM109 | OeREM1 | OeREM109 | 0.0000 | 0.0000 | 0.0000 | 0.0000 | 0.0000 | 0.0000 | 0.0000 | 0.0000 | 0.0000 | 0.0000 |
| OeREM1-OeREM121 | OeREM1 | OeREM121 | 0.0000 | 0.0000 | 0.0000 | 0.0000 | 0.0000 | 0.0000 | 0.0000 | 0.0000 | 0.0000 | 0.0000 |
| OeREM2-OeREM11 | OeREM2 | OeREM11 | 15.3643 | 13.1023 | 13.7003 | 18.0500 | 17.1130 | 0.0140 | 0.4713 | 0.0153 | 0.1840 | 0.5750 |
| OeREM2-OeREM84 | OeREM2 | OeREM84 | 15.3643 | 13.1023 | 13.7003 | 18.0500 | 17.1130 | 13.1757 | 20.1220 | 14.5640 | 32.6660 | 34.7290 |
| OeARF2-OeARF11 | OeARF2 | OeARF11 | 29.4437 | 16.3783 | 13.1817 | 71.2000 | 48.2400 | 10.2323 | 26.5940 | 11.2463 | 43.0550 | 30.4610 |
| OeARF2-OeARF34 | OeARF2 | OeARF34 | 29.4437 | 16.3783 | 13.1817 | 71.2000 | 48.2400 | 2.4890 | 1.7680 | 1.0727 | 3.4470 | 3.6870 |
| OeARF3-OeARF22 | OeARF3 | OeARF22 | 0.6643 | 5.6840 | 0.8883 | 38.6470 | 7.7800 | 2.5040 | 27.9307 | 12.6463 | 69.5790 | 54.5350 |
| OeARF3-OeARF33 | OeARF3 | OeARF33 | 0.6643 | 5.6840 | 0.8883 | 38.6470 | 7.7800 | 4.7353 | 21.3410 | 4.7173 | 35.3480 | 35.9960 |
| OeREM6-OeREM82 | OeREM6 | OeREM82 | 0.0000 | 0.0000 | 0.0000 | 0.0000 | 0.0000 | 0.0000 | 0.0000 | 0.0000 | 0.0000 | 0.0000 |
| OeARF4-OeARF31 | OeARF4 | OeARF31 | 7.5983 | 6.7993 | 4.4847 | 61.3810 | 135.2560 | 6.1710 | 10.5647 | 8.5740 | 19.1980 | 73.6610 |
| OeREM11-OeREM16 | OeREM11 | OeREM16 | 0.0140 | 0.4713 | 0.0153 | 0.1840 | 0.5750 | 24.1867 | 14.0677 | 7.8917 | 7.9220 | 6.5790 |
| OeREM11-OeREM74 | OeREM11 | OeREM74 | 0.0140 | 0.4713 | 0.0153 | 0.1840 | 0.5750 | 12.0413 | 14.1863 | 8.9923 | 13.2070 | 17.8550 |
| OeREM11-OeREM84 | OeREM11 | OeREM84 | 0.0140 | 0.4713 | 0.0153 | 0.1840 | 0.5750 | 13.1757 | 20.1220 | 14.5640 | 32.6660 | 34.7290 |
| OeARF5-OeARF27 | OeARF5 | OeARF27 | 0.6567 | 0.1057 | 0.8077 | 10.4160 | 15.0100 | 0.1393 | 1.5720 | 0.4567 | 17.0650 | 21.8910 |
| OeARF5-OeARF29 | OeARF5 | OeARF29 | 0.6567 | 0.1057 | 0.8077 | 10.4160 | 15.0100 | 0.0243 | 0.0330 | 0.0223 | 0.1330 | 0.0590 |
| OeREM12-OeREM63 | OeREM12 | OeREM63 | 7.9083 | 7.0397 | 2.2980 | 2.6210 | 2.1270 | 0.0000 | 0.0000 | 0.0000 | 0.0000 | 0.0000 |
| OeARF6-OeARF28 | OeARF6 | OeARF28 | 21.7167 | 53.7577 | 59.0350 | 112.5960 | 62.8780 | 17.3340 | 34.8483 | 26.2720 | 68.4490 | 32.0160 |
| OeREM13-OeREM56 | OeREM13 | OeREM56 | 0.3500 | 0.2530 | 0.8400 | 2.5700 | 2.1140 | 3.6303 | 0.0000 | 0.0253 | 0.0000 | 0.1350 |
| OeARF7-OeARF27 | OeARF7 | OeARF27 | 1.6543 | 2.1843 | 0.5847 | 1.8930 | 1.1310 | 0.1393 | 1.5720 | 0.4567 | 17.0650 | 21.8910 |
| OeLAV1-OeLAV5 | OeLAV1 | OeLAV5 | 0.0000 | 0.0000 | 0.0000 | 0.0000 | 0.0000 | 0.0000 | 0.0000 | 0.0000 | 0.0000 | 0.0000 |
| OeHSI1-OeHSI4 | OeHSI1 | OeHSI4 | 0.0093 | 3.3030 | 5.1410 | 2.2420 | 2.8040 | 1.0700 | 4.5167 | 5.5670 | 5.5790 | 6.2340 |
| OeHSI1-OeHSI6 | OeHSI1 | OeHSI6 | 0.0093 | 3.3030 | 5.1410 | 2.2420 | 2.8040 | 5.5297 | 14.0987 | 22.1550 | 17.0280 | 26.1800 |
| OeHSI1-OeHSI9 | OeHSI1 | OeHSI9 | 0.0093 | 3.3030 | 5.1410 | 2.2420 | 2.8040 | 6.7343 | 10.9140 | 13.8143 | 36.0420 | 15.7550 |
| OeARF8-OeARF39 | OeARF8 | OeARF39 | 5.0947 | 8.5013 | 4.6697 | 8.9620 | 3.1320 | 0.0427 | 3.1940 | 0.3500 | 4.9330 | 6.1790 |
| OeARF8-OeARF43 | OeARF8 | OeARF43 | 5.0947 | 8.5013 | 4.6697 | 8.9620 | 3.1320 | 8.0597 | 14.3083 | 13.5510 | 20.2860 | 8.4370 |
| OeARF9-OeARF20 | OeARF9 | OeARF20 | 9.9343 | 28.1633 | 22.5143 | 107.8810 | 67.5400 | 0.1710 | 1.0287 | 0.0000 | 0.0610 | 0.3780 |
| OeARF9-OeARF44 | OeARF9 | OeARF44 | 9.9343 | 28.1633 | 22.5143 | 107.8810 | 67.5400 | 3.5530 | 6.9537 | 8.0680 | 15.3840 | 9.9000 |
| OeARF10-OeARF36 | OeARF10 | OeARF36 | 0.1433 | 7.6150 | 4.3263 | 13.9160 | 16.5800 | 0.0080 | 2.2770 | 0.2250 | 9.7690 | 6.4850 |
| OeARF10-OeARF45 | OeARF10 | OeARF45 | 0.1433 | 7.6150 | 4.3263 | 13.9160 | 16.5800 | 0.0000 | 0.7963 | 0.0000 | 0.0210 | 0.0180 |
| OeREM16-OeREM71 | OeREM16 | OeREM71 | 24.1867 | 14.0677 | 7.8917 | 7.9220 | 6.5790 | 0.1863 | 6.6780 | 0.6977 | 2.7670 | 3.5230 |
| OeREM16-OeREM74 | OeREM16 | OeREM74 | 24.1867 | 14.0677 | 7.8917 | 7.9220 | 6.5790 | 12.0413 | 14.1863 | 8.9923 | 13.2070 | 17.8550 |
| OeREM20-OeREM102 | OeREM20 | OeREM102 | 0.0000 | 0.0000 | 0.0000 | 0.0000 | 0.0000 | 0.0000 | 0.0000 | 0.0000 | 0.0000 | 0.0000 |
| OeREM21-OeREM91 | OeREM21 | OeREM91 | 0.0000 | 0.0440 | 0.0000 | 0.0000 | 0.0000 | 0.0000 | 0.0000 | 0.0000 | 0.0000 | 0.0000 |
| OeRAV3-OeRAV4 | OeRAV3 | OeRAV4 | 0.5800 | 11.8660 | 105.6843 | 46.8250 | 30.6950 | 0.2657 | 2.0250 | 2.9537 | 15.3620 | 6.9520 |
| OeARF11-OeARF34 | OeARF11 | OeARF34 | 10.2323 | 26.5940 | 11.2463 | 43.0550 | 30.4610 | 2.4890 | 1.7680 | 1.0727 | 3.4470 | 3.6870 |
| OeREM28-OeREM71 | OeREM28 | OeREM71 | 0.2373 | 0.5563 | 0.0000 | 0.1840 | 0.1230 | 0.1863 | 6.6780 | 0.6977 | 2.7670 | 3.5230 |
| OeARF12-OeARF26 | OeARF12 | OeARF26 | 0.6320 | 1.8633 | 0.3523 | 0.7360 | 1.7180 | 0.0740 | 1.0310 | 1.0477 | 1.3800 | 2.8170 |
| OeARF13-OeARF14 | OeARF13 | OeARF14 | 1.6457 | 3.7610 | 0.8493 | 0.4440 | 1.4400 | 0.0637 | 0.1780 | 0.0270 | 0.0680 | 0.1810 |
| OeARF13-OeARF26 | OeARF13 | OeARF26 | 1.6457 | 3.7610 | 0.8493 | 0.4440 | 1.4400 | 0.0740 | 1.0310 | 1.0477 | 1.3800 | 2.8170 |
| OeHSI2-OeHSI5 | OeHSI2 | OeHSI5 | 4.5047 | 20.6863 | 28.4277 | 41.4080 | 51.2180 | 2.5753 | 8.3060 | 6.6460 | 8.4910 | 32.7760 |
| OeRAV5-OeRAV9 | OeRAV5 | OeRAV9 | 0.0000 | 0.0000 | 0.0000 | 0.1400 | 0.0000 | 1.0613 | 4.6103 | 9.5373 | 7.9530 | 37.3030 |
| OeREM33-OeREM42 | OeREM33 | OeREM42 | 0.0000 | 0.0000 | 0.0207 | 0.0000 | 0.0000 | 0.0000 | 0.0000 | 0.0000 | 0.0000 | 0.0000 |
| OeRAV7-OeRAV8 | OeRAV7 | OeRAV8 | 0.0157 | 2.9470 | 0.7903 | 2.6720 | 3.4510 | 13.0213 | 0.1200 | 0.2213 | 0.0340 | 0.2430 |
| OeRAV7-OeRAV10 | OeRAV7 | OeRAV10 | 0.0157 | 2.9470 | 0.7903 | 2.6720 | 3.4510 | 0.0140 | 14.1097 | 5.4997 | 2.0620 | 0.5680 |
| OeRAV7-OeRAV12 | OeRAV7 | OeRAV12 | 0.0157 | 2.9470 | 0.7903 | 2.6720 | 3.4510 | 1.1307 | 8.1010 | 13.6440 | 3.4320 | 7.9230 |
| OeRAV7-OeRAV14 | OeRAV7 | OeRAV14 | 0.0157 | 2.9470 | 0.7903 | 2.6720 | 3.4510 | 0.0000 | 2.8363 | 0.0247 | 0.1480 | 0.3290 |
| OeARF16-OeARF22 | OeARF16 | OeARF22 | 10.0533 | 14.4990 | 27.9823 | 71.8920 | 75.9950 | 2.5040 | 27.9307 | 12.6463 | 69.5790 | 54.5350 |
| OeARF16-OeARF33 | OeARF16 | OeARF33 | 10.0533 | 14.4990 | 27.9823 | 71.8920 | 75.9950 | 4.7353 | 21.3410 | 4.7173 | 35.3480 | 35.9960 |
| OeARF17-OeARF47 | OeARF17 | OeARF47 | 2.8707 | 17.3593 | 14.5340 | 43.2440 | 24.2370 | 121.1360 | 87.9693 | 161.6740 | 209.9700 | 166.7340 |
| OeREM37-OeREM51 | OeREM37 | OeREM51 | 0.0000 | 0.0000 | 0.0000 | 0.0000 | 0.0000 | 0.0000 | 0.0000 | 0.0000 | 0.0000 | 0.0000 |
| OeRAV8-OeRAV10 | OeRAV8 | OeRAV10 | 13.0213 | 0.1200 | 0.2213 | 0.0340 | 0.2430 | 0.0140 | 14.1097 | 5.4997 | 2.0620 | 0.5680 |
| OeRAV8-OeRAV12 | OeRAV8 | OeRAV12 | 13.0213 | 0.1200 | 0.2213 | 0.0340 | 0.2430 | 1.1307 | 8.1010 | 13.6440 | 3.4320 | 7.9230 |
| OeRAV8-OeRAV14 | OeRAV8 | OeRAV14 | 13.0213 | 0.1200 | 0.2213 | 0.0340 | 0.2430 | 0.0000 | 2.8363 | 0.0247 | 0.1480 | 0.3290 |
| OeARF19-OeARF20 | OeARF19 | OeARF20 | 0.0000 | 0.1223 | 0.0000 | 0.0260 | 0.4550 | 0.1710 | 1.0287 | 0.0000 | 0.0610 | 0.3780 |
| OeARF19-OeARF32 | OeARF19 | OeARF32 | 0.0000 | 0.1223 | 0.0000 | 0.0260 | 0.4550 | 3.8073 | 11.6140 | 11.5753 | 12.4530 | 7.9000 |
| OeARF19-OeARF38 | OeARF19 | OeARF38 | 0.0000 | 0.1223 | 0.0000 | 0.0260 | 0.4550 | 2.1793 | 6.0260 | 14.3180 | 34.9250 | 15.4470 |
| OeARF19-OeARF43 | OeARF19 | OeARF43 | 0.0000 | 0.1223 | 0.0000 | 0.0260 | 0.4550 | 8.0597 | 14.3083 | 13.5510 | 20.2860 | 8.4370 |
| OeARF20-OeARF24 | OeARF20 | OeARF24 | 0.1710 | 1.0287 | 0.0000 | 0.0610 | 0.3780 | 0.6607 | 0.7390 | 0.1130 | 0.0800 | 0.1250 |
| OeARF20-OeARF39 | OeARF20 | OeARF39 | 0.1710 | 1.0287 | 0.0000 | 0.0610 | 0.3780 | 0.0427 | 3.1940 | 0.3500 | 4.9330 | 6.1790 |
| OeARF20-OeARF43 | OeARF20 | OeARF43 | 0.1710 | 1.0287 | 0.0000 | 0.0610 | 0.3780 | 8.0597 | 14.3083 | 13.5510 | 20.2860 | 8.4370 |
| OeHSI4-OeHSI6 | OeHSI4 | OeHSI6 | 1.0700 | 4.5167 | 5.5670 | 5.5790 | 6.2340 | 5.5297 | 14.0987 | 22.1550 | 17.0280 | 26.1800 |
| OeRAV10-OeRAV12 | OeRAV10 | OeRAV12 | 0.0140 | 14.1097 | 5.4997 | 2.0620 | 0.5680 | 1.1307 | 8.1010 | 13.6440 | 3.4320 | 7.9230 |
| OeARF24-OeARF39 | OeARF24 | OeARF39 | 0.6607 | 0.7390 | 0.1130 | 0.0800 | 0.1250 | 0.0427 | 3.1940 | 0.3500 | 4.9330 | 6.1790 |
| OeARF25-OeARF36 | OeARF25 | OeARF36 | 0.3453 | 17.0707 | 5.3767 | 37.8820 | 17.5070 | 0.0080 | 2.2770 | 0.2250 | 9.7690 | 6.4850 |
| OeRAV11-OeRAV13 | OeRAV11 | OeRAV13 | 0.6377 | 12.4133 | 16.4290 | 0.0000 | 0.4700 | 0.1093 | 2.6857 | 0.3563 | 0.2490 | 0.4040 |
| OeLAV2-OeLAV3 | OeLAV2 | OeLAV3 | 0.0000 | 0.1103 | 0.0000 | 0.0000 | 0.0000 | 0.0000 | 1.0527 | 0.0000 | 2.1590 | 0.5960 |
| OeLAV2-OeLAV4 | OeLAV2 | OeLAV4 | 0.0000 | 0.1103 | 0.0000 | 0.0000 | 0.0000 | 0.0000 | 0.0000 | 0.0000 | 0.0000 | 0.0000 |
| OeARF27-OeARF29 | OeARF27 | OeARF29 | 0.1393 | 1.5720 | 0.4567 | 17.0650 | 21.8910 | 0.0243 | 0.0330 | 0.0223 | 0.1330 | 0.0590 |
| OeREM56-OeREM57 | OeREM56 | OeREM57 | 3.6303 | 0.0000 | 0.0253 | 0.0000 | 0.1350 | 3.9440 | 7.4950 | 7.2220 | 7.5480 | 6.2210 |
| OeREM58-OeREM59 | OeREM58 | OeREM59 | 0.0000 | 0.0000 | 0.0000 | 0.0000 | 0.0000 | 0.0000 | 0.0000 | 0.0000 | 0.0000 | 0.0000 |
| OeREM59-OeREM60 | OeREM59 | OeREM60 | 0.0000 | 0.0000 | 0.0000 | 0.0000 | 0.0000 | 0.0000 | 0.0000 | 0.0000 | 0.0000 | 0.0000 |
| OeREM61-OeREM62 | OeREM61 | OeREM62 | 0.0000 | 0.0000 | 0.0000 | 0.0000 | 0.0000 | 0.0000 | 0.0000 | 0.0000 | 0.0000 | 0.0000 |
| OeREM64-OeREM111 | OeREM64 | OeREM111 | 0.0000 | 0.0000 | 0.0000 | 0.0000 | 0.0000 | 0.0000 | 0.0000 | 0.0000 | 0.0000 | 0.0000 |
| OeREM64-OeREM116 | OeREM64 | OeREM116 | 0.0000 | 0.0000 | 0.0000 | 0.0000 | 0.0000 | 0.0000 | 0.0000 | 0.0000 | 0.0000 | 0.0000 |
| OeREM69-OeREM70 | OeREM69 | OeREM70 | 3.3123 | 3.5233 | 2.2683 | 14.3770 | 15.1620 | 15.3850 | 10.9443 | 5.6593 | 10.7710 | 12.0900 |
| OeREM71-OeREM74 | OeREM71 | OeREM74 | 0.1863 | 6.6780 | 0.6977 | 2.7670 | 3.5230 | 12.0413 | 14.1863 | 8.9923 | 13.2070 | 17.8550 |
| OeREM72-OeREM73 | OeREM72 | OeREM73 | 1.0460 | 0.7860 | 0.6920 | 3.4490 | 1.9560 | 7.3970 | 2.7320 | 1.7997 | 7.5580 | 9.5590 |
| OeREM74-OeREM75 | OeREM74 | OeREM75 | 12.0413 | 14.1863 | 8.9923 | 13.2070 | 17.8550 | 0.0000 | 2.5433 | 0.0170 | 0.3830 | 0.8870 |
| OeARF30-OeARF40 | OeARF30 | OeARF40 | 73.5130 | 83.4973 | 36.0377 | 226.8460 | 152.3690 | 12.2203 | 9.1453 | 9.7200 | 32.9600 | 50.7630 |
| OeARF32-OeARF38 | OeARF32 | OeARF38 | 3.8073 | 11.6140 | 11.5753 | 12.4530 | 7.9000 | 2.1793 | 6.0260 | 14.3180 | 34.9250 | 15.4470 |
| OeRAV12-OeRAV14 | OeRAV12 | OeRAV14 | 1.1307 | 8.1010 | 13.6440 | 3.4320 | 7.9230 | 0.0000 | 2.8363 | 0.0247 | 0.1480 | 0.3290 |
| OeLAV3-OeLAV4 | OeLAV3 | OeLAV4 | 0.0000 | 1.0527 | 0.0000 | 2.1590 | 0.5960 | 0.0000 | 0.0000 | 0.0000 | 0.0000 | 0.0000 |
| OeARF35-OeARF38 | OeARF35 | OeARF38 | 5.4790 | 12.5437 | 2.1763 | 10.5180 | 15.3940 | 2.1793 | 6.0260 | 14.3180 | 34.9250 | 15.4470 |
| OeARF36-OeARF45 | OeARF36 | OeARF45 | 0.0080 | 2.2770 | 0.2250 | 9.7690 | 6.4850 | 0.0000 | 0.7963 | 0.0000 | 0.0210 | 0.0180 |
| OeREM92-OeREM93 | OeREM92 | OeREM93 | 0.0000 | 0.1767 | 0.0000 | 0.0000 | 0.0000 | 0.0000 | 0.2253 | 0.0000 | 0.0000 | 0.0000 |
| OeHSI6-OeHSI9 | OeHSI6 | OeHSI9 | 5.5297 | 14.0987 | 22.1550 | 17.0280 | 26.1800 | 6.7343 | 10.9140 | 13.8143 | 36.0420 | 15.7550 |
| OeARF39-OeARF43 | OeARF39 | OeARF43 | 0.0427 | 3.1940 | 0.3500 | 4.9330 | 6.1790 | 8.0597 | 14.3083 | 13.5510 | 20.2860 | 8.4370 |
| OeREM103-OeREM104 | OeREM103 | OeREM104 | 0.0000 | 0.0000 | 0.0000 | 0.0450 | 0.0000 | 0.0000 | 0.0000 | 0.0000 | 0.0000 | 0.0000 |
| OeARF48-OeARF49 | OeARF48 | OeARF49 | 1.3620 | 1.9157 | 3.0093 | 0.1430 | 0.1270 | 0.1297 | 0.0097 | 0.0077 | 0.0000 | 0.0000 |
| OeREM109-OeREM121 | OeREM109 | OeREM121 | 0.0000 | 0.0000 | 0.0000 | 0.0000 | 0.0000 | 0.0000 | 0.0000 | 0.0000 | 0.0000 | 0.0000 |
| OeREM110-OeREM120 | OeREM110 | OeREM120 | 0.0000 | 0.0000 | 0.0000 | 0.0000 | 0.0000 | 0.0000 | 0.0000 | 0.0000 | 0.0000 | 0.0000 |
| OeREM111-OeREM116 | OeREM111 | OeREM116 | 0.0000 | 0.0000 | 0.0000 | 0.0000 | 0.0000 | 0.0000 | 0.0000 | 0.0000 | 0.0000 | 0.0000 |
| OeREM112-OeREM117 | OeREM112 | OeREM117 | 0.0000 | 0.0000 | 0.0000 | 0.0000 | 0.0000 | 0.0000 | 0.0000 | 0.0000 | 0.0000 | 0.0000 |
| OeREM113-OeREM118 | OeREM113 | OeREM118 | 0.0000 | 0.0000 | 0.0000 | 0.0000 | 0.0000 | 0.0000 | 0.0000 | 0.0000 | 0.0000 | 0.0000 |

**Table S8. The Ka/Ks ratios of duplicated genes of B3 superfamily in olive**

| **Gene Name Pairs** | **Gene ID Pair** | **Method** | **Ka** | **Ks** | **Ka/Ks** | **P-Value(Fisher)** |
| --- | --- | --- | --- | --- | --- | --- |
| OeARF1-OeARF23 | GWHGAOPM000403-GWHGAOPM024600 | MA | 0.0971 | 0.2148 | 0.4520 | 6.60E-11 |
| OeRAV2-OeRAV3 | GWHGAOPM001565-GWHGAOPM010989 | MA | 1.0440 | 0.8577 | 1.2172 | 1.33E-05 |
| OeRAV2-OeRAV4 | GWHGAOPM001565-GWHGAOPM015562 | MA | 1.0217 | 0.9285 | 1.1004 | 0.0302474 |
| OeREM1-OeREM109 | GWHGAOPM002123-GWHGAOPM049255 | MA | 0.9734 | 1.1001 | 0.8848 | 0.0457466 |
| OeREM1-OeREM121 | GWHGAOPM002123-GWHGAOPM052522 | MA | NA | NA | NA | NA |
| OeREM2-OeREM11 | GWHGAOPM002258-GWHGAOPM004396 | MA | 0.5147 | 2.5975 | 0.1982 | 0 |
| OeREM2-OeREM84 | GWHGAOPM002258-GWHGAOPM036972 | MA | 0.0577 | 0.3128 | 0.1843 | 3.18E-22 |
| OeARF2-OeARF11 | GWHGAOPM002508-GWHGAOPM013510 | MA | 1.0161 | 0.9387 | 1.0824 | 0.0179138 |
| OeARF2-OeARF34 | GWHGAOPM002508-GWHGAOPM037318 | MA | 0.9956 | 1.0162 | 0.9797 | 0.510504 |
| OeARF3-OeARF22 | GWHGAOPM003114-GWHGAOPM023961 | MA | 0.8669 | 0.9027 | 0.9604 | 0.360472 |
| OeARF3-OeARF33 | GWHGAOPM003114-GWHGAOPM036308 | MA | 1.0136 | 0.9529 | 1.0637 | 0.0152306 |
| OeREM6-OeREM82 | GWHGAOPM003214-GWHGAOPM035626 | MA | 0.9420 | 1.2272 | 0.7676 | 7.63E-06 |
| OeARF4-OeARF31 | GWHGAOPM003954-GWHGAOPM035738 | MA | 0.9903 | 1.0356 | 0.9563 | 0.100559 |
| OeREM11-OeREM16 | GWHGAOPM004396-GWHGAOPM008419 | MA | 0.6232 | 2.4117 | 0.2584 | 0 |
| OeREM11-OeREM74 | GWHGAOPM004396-GWHGAOPM033247 | MA | 0.6124 | 2.4441 | 0.2506 | 0 |
| OeREM11-OeREM84 | GWHGAOPM004396-GWHGAOPM036972 | MA | 0.5009 | 2.3197 | 0.2159 | 0 |
| OeARF5-OeARF27 | GWHGAOPM005137-GWHGAOPM030978 | MA | 1.0504 | 0.8190 | 1.2826 | 4.93E-05 |
| OeARF5-OeARF29 | GWHGAOPM005137-GWHGAOPM032112 | MA | 0.9881 | 1.0459 | 0.9447 | 0.0896701 |
| OeREM12-OeREM63 | GWHGAOPM005461-GWHGAOPM031741 | MA | 0.9813 | 1.0693 | 0.9177 | 0.20653 |
| OeARF6-OeARF28 | GWHGAOPM005523-GWHGAOPM031630 | MA | 0.9971 | 1.0108 | 0.9865 | 0.696867 |
| OeREM13-OeREM56 | GWHGAOPM005686-GWHGAOPM031386 | MA | 1.0143 | 0.9452 | 1.0731 | 0.206705 |
| OeARF7-OeARF27 | GWHGAOPM006011-GWHGAOPM030978 | MA | 0.9941 | 1.0241 | 0.9707 | 0.476029 |
| OeLAV1-OeLAV5 | GWHGAOPM006235-GWHGAOPM045268 | MA | 0.0796 | 0.3361 | 0.2367 | 2.20E-25 |
| OeHSI1-OeHSI4 | GWHGAOPM006368-GWHGAOPM022959 | MA | 0.2839 | 0.9612 | 0.2954 | 3.15E-73 |
| OeHSI1-OeHSI6 | GWHGAOPM006368-GWHGAOPM043066 | MA | 0.9669 | 1.1252 | 0.8593 | 2.84E-07 |
| OeHSI1-OeHSI9 | GWHGAOPM006368-GWHGAOPM045408 | MA | 0.1258 | 0.3624 | 0.3470 | 1.88E-23 |
| OeARF8-OeARF39 | GWHGAOPM006478-GWHGAOPM043409 | MA | 0.9771 | 1.0895 | 0.8969 | 0.0037129 |
| OeARF8-OeARF43 | GWHGAOPM006478-GWHGAOPM045502 | MA | 0.9865 | 1.0517 | 0.9380 | 0.0564058 |
| OeARF9-OeARF20 | GWHGAOPM006492-GWHGAOPM022620 | MA | 0.5991 | 2.5416 | 0.2357 | 0 |
| OeARF9-OeARF44 | GWHGAOPM006492-GWHGAOPM045522 | MA | 0.0566 | 0.2386 | 0.2371 | 4.54E-18 |
| OeARF10-OeARF36 | GWHGAOPM007921-GWHGAOPM040846 | MA | 0.9945 | 1.0228 | 0.9724 | 0.445108 |
| OeARF10-OeARF45 | GWHGAOPM007921-GWHGAOPM046357 | MA | 0.9774 | 1.0797 | 0.9053 | 0.0028715 |
| OeREM16-OeREM71 | GWHGAOPM008419-GWHGAOPM032601 | MA | 0.3119 | 0.8667 | 0.3598 | 1.93E-19 |
| OeREM16-OeREM74 | GWHGAOPM008419-GWHGAOPM033247 | MA | 0.1048 | 0.2472 | 0.4241 | 9.91E-06 |
| OeREM20-OeREM102 | GWHGAOPM009492-GWHGAOPM047351 | MA | 0.9488 | 1.2087 | 0.7850 | 0.0004175 |
| OeREM21-OeREM91 | GWHGAOPM010206-GWHGAOPM040171 | MA | 1.0154 | 0.9452 | 1.0743 | 0.379205 |
| OeRAV3-OeRAV4 | GWHGAOPM010989-GWHGAOPM015562 | MA | 0.0397 | 0.2703 | 0.1471 | 1.01E-19 |
| OeARF11-OeARF34 | GWHGAOPM013510-GWHGAOPM037318 | MA | 0.2016 | 0.8396 | 0.2401 | 6.75E-86 |
| OeREM28-OeREM71 | GWHGAOPM013665-GWHGAOPM032601 | MA | 0.9625 | 1.1418 | 0.8430 | 0.0121787 |
| OeARF12-OeARF26 | GWHGAOPM013812-GWHGAOPM029659 | MA | 0.9832 | 1.0655 | 0.9228 | 0.0048424 |
| OeARF13-OeARF14 | GWHGAOPM013821-GWHGAOPM013822 | MA | 1.0088 | 0.9664 | 1.0440 | 0.175781 |
| OeARF13-OeARF26 | GWHGAOPM013821-GWHGAOPM029659 | MA | 0.9909 | 1.0362 | 0.9563 | 0.127556 |
| OeHSI2-OeHSI5 | GWHGAOPM015868-GWHGAOPM029166 | MA | 0.9895 | 1.0407 | 0.9508 | 0.0711821 |
| OeRAV5-OeRAV9 | GWHGAOPM016934-GWHGAOPM023375 | MA | 0.9796 | 1.0726 | 0.9134 | 0.0497975 |
| OeREM33-OeREM42 | GWHGAOPM017025-GWHGAOPM023881 | MA | 1.0108 | 0.9588 | 1.0542 | 0.280238 |
| OeRAV7-OeRAV8 | GWHGAOPM017295-GWHGAOPM021666 | MA | 1.0167 | 0.9437 | 1.0774 | 0.212012 |
| OeRAV7-OeRAV10 | GWHGAOPM017295-GWHGAOPM023559 | MA | 1.0125 | 0.9550 | 1.0602 | 0.27114 |
| OeRAV7-OeRAV12 | GWHGAOPM017295-GWHGAOPM036211 | MA | 0.9755 | 1.0863 | 0.8980 | 0.0259204 |
| OeRAV7-OeRAV14 | GWHGAOPM017295-GWHGAOPM042688 | MA | 0.9725 | 1.0976 | 0.8860 | 0.0367522 |
| OeARF16-OeARF22 | GWHGAOPM017389-GWHGAOPM023961 | MA | 0.5213 | 0.4081 | 1.2775 | 0.0001933 |
| OeARF16-OeARF33 | GWHGAOPM017389-GWHGAOPM036308 | MA | 1.0071 | 0.9736 | 1.0344 | 0.202278 |
| OeARF17-OeARF47 | GWHGAOPM017781-GWHGAOPM048473 | MA | 0.9837 | 1.0649 | 0.9237 | 0.0160194 |
| OeREM37-OeREM51 | GWHGAOPM019360-GWHGAOPM028749 | MA | 0.9749 | 1.0894 | 0.8950 | 0.142553 |
| OeRAV8-OeRAV10 | GWHGAOPM021666-GWHGAOPM023559 | MA | 0.2567 | 1.0894 | 0.2356 | 1.89E-24 |
| OeRAV8-OeRAV12 | GWHGAOPM021666-GWHGAOPM036211 | MA | 0.9730 | 1.1187 | 0.8698 | 0.0381704 |
| OeRAV8-OeRAV14 | GWHGAOPM021666-GWHGAOPM042688 | MA | 0.9547 | 1.1637 | 0.8204 | 0.0020152 |
| OeARF19-OeARF20 | GWHGAOPM021778-GWHGAOPM022620 | MA | 0.3735 | 3.1245 | 0.1195 | 0 |
| OeARF19-OeARF32 | GWHGAOPM021778-GWHGAOPM036122 | MA | 0.9738 | 1.1010 | 0.8845 | 0.0003322 |
| OeARF19-OeARF38 | GWHGAOPM021778-GWHGAOPM042595 | MA | 1.0065 | 0.9762 | 1.0310 | 0.374515 |
| OeARF19-OeARF43 | GWHGAOPM021778-GWHGAOPM045502 | MA | 0.3402 | 3.3242 | 0.1023 | 0 |
| OeARF20-OeARF24 | GWHGAOPM022620-GWHGAOPM025413 | MA | 0.2075 | 0.7889 | 0.2631 | 9.64E-57 |
| OeARF20-OeARF39 | GWHGAOPM022620-GWHGAOPM043409 | MA | 1.0170 | 0.9389 | 1.0832 | 0.0199114 |
| OeARF20-OeARF43 | GWHGAOPM022620-GWHGAOPM045502 | MA | 0.1699 | 0.9771 | 0.1739 | 2.88E-110 |
| OeHSI4-OeHSI6 | GWHGAOPM022959-GWHGAOPM043066 | MA | 0.9842 | 1.0592 | 0.9292 | 0.0101985 |
| OeRAV10-OeRAV12 | GWHGAOPM023559-GWHGAOPM036211 | MA | 1.0144 | 0.9504 | 1.0673 | 0.238375 |
| OeARF24-OeARF39 | GWHGAOPM025413-GWHGAOPM043409 | MA | 0.9894 | 1.0396 | 0.9517 | 0.171761 |
| OeARF25-OeARF36 | GWHGAOPM028413-GWHGAOPM040846 | MA | 0.9913 | 1.0360 | 0.9569 | 0.193804 |
| OeRAV11-OeRAV13 | GWHGAOPM028473-GWHGAOPM040896 | MA | 0.1280 | 0.2998 | 0.4270 | 1.92E-08 |
| OeLAV2-OeLAV3 | GWHGAOPM030431-GWHGAOPM036728 | MA | 0.9695 | 1.1097 | 0.8737 | 0.0282429 |
| OeLAV2-OeLAV4 | GWHGAOPM030431-GWHGAOPM039592 | MA | 1.0338 | 0.8829 | 1.1709 | 0.0080424 |
| OeARF27-OeARF29 | GWHGAOPM030978-GWHGAOPM032112 | MA | 0.9879 | 1.0482 | 0.9424 | 0.10479 |
| OeREM56-OeREM57 | GWHGAOPM031386-GWHGAOPM031387 | MA | 1.0133 | 0.9518 | 1.0646 | 0.283503 |
| OeREM58-OeREM59 | GWHGAOPM031695-GWHGAOPM031696 | MA | 0.0363 | 0.0581 | 0.6238 | 0.147591 |
| OeREM59-OeREM60 | GWHGAOPM031696-GWHGAOPM031697 | MA | 0.0361 | 0.0675 | 0.5341 | 0.0705662 |
| OeREM61-OeREM62 | GWHGAOPM031699-GWHGAOPM031700 | MA | 0.0512 | 0.0830 | 0.6173 | 0.105694 |
| OeREM64-OeREM111 | GWHGAOPM031977-GWHGAOPM049749 | MA | 0.9526 | 1.1890 | 0.8012 | 0.0026584 |
| OeREM64-OeREM116 | GWHGAOPM031977-GWHGAOPM050943 | MA | 0.9526 | 1.1890 | 0.8012 | 0.0026584 |
| OeREM69-OeREM70 | GWHGAOPM032590-GWHGAOPM032591 | MA | 0.4515 | 0.6504 | 0.6943 | 0.0034038 |
| OeREM71-OeREM74 | GWHGAOPM032601-GWHGAOPM033247 | MA | 0.2679 | 0.9645 | 0.2778 | 4.86E-29 |
| OeREM72-OeREM73 | GWHGAOPM032603-GWHGAOPM032604 | MA | 0.4222 | 0.5387 | 0.7838 | 0.0636097 |
| OeREM74-OeREM75 | GWHGAOPM033247-GWHGAOPM033248 | MA | 0.6246 | 2.5056 | 0.2493 | 0 |
| OeARF30-OeARF40 | GWHGAOPM033730-GWHGAOPM044039 | MA | 1.0011 | 0.9958 | 1.0053 | 0.817978 |
| OeARF32-OeARF38 | GWHGAOPM036122-GWHGAOPM042595 | MA | 1.0185 | 0.9318 | 1.0931 | 0.0126175 |
| OeRAV12-OeRAV14 | GWHGAOPM036211-GWHGAOPM042688 | MA | 0.9873 | 1.0470 | 0.9430 | 0.25358 |
| OeLAV3-OeLAV4 | GWHGAOPM036728-GWHGAOPM039592 | MA | 1.0181 | 0.9382 | 1.0852 | 0.201875 |
| OeARF35-OeARF38 | GWHGAOPM040448-GWHGAOPM042595 | MA | 1.0131 | 0.9561 | 1.0596 | 0.108135 |
| OeARF36-OeARF45 | GWHGAOPM040846-GWHGAOPM046357 | MA | 0.9924 | 1.0272 | 0.9660 | 0.381056 |
| OeREM92-OeREM93 | GWHGAOPM041635-GWHGAOPM041636 | MA | 0.9767 | 1.0836 | 0.9013 | 0.0902123 |
| OeHSI6-OeHSI9 | GWHGAOPM043066-GWHGAOPM045408 | MA | 0.9889 | 1.0414 | 0.9496 | 0.0827393 |
| OeARF39-OeARF43 | GWHGAOPM043409-GWHGAOPM045502 | MA | 1.0178 | 0.9348 | 1.0888 | 0.0186058 |
| OeREM103-OeREM104 | GWHGAOPM047827-GWHGAOPM047828 | MA | 0.1519 | 0.3776 | 0.4023 | 3.81E-09 |
| OeARF48-OeARF49 | GWHGAOPM048959-GWHGAOPM052974 | MA | 0.0510 | 0.0647 | 0.7878 | 0.197304 |
| OeREM109-OeREM121 | GWHGAOPM049255-GWHGAOPM052522 | MA | 0.9734 | 1.1001 | 0.8848 | 0.0457466 |
| OeREM110-OeREM120 | GWHGAOPM049556-GWHGAOPM052256 | MA | 0.9193 | 1.3788 | 0.6667 | 1.54E-15 |
| OeREM111-OeREM116 | GWHGAOPM049749-GWHGAOPM050943 | MA | NA | NA | NA | NA |
| OeREM112-OeREM117 | GWHGAOPM049750-GWHGAOPM050944 | MA | NA | NA | NA | NA |
| OeREM113-OeREM118 | GWHGAOPM049755-GWHGAOPM050955 | MA | NA | NA | NA | NA |
