# Supplementary figures and images for: Genome-Wide Analysis of the Molecular Functions of B3 Superfamily in Oil Biosynthesis in Olive (Olea europaea L.)
Source: Biomed Res Int. 2023 Feb 14;2023:6051511. doi: 10.1155/2023/6051511 (PMC9943606; doi:10.1155/2023/6051511)

**b**

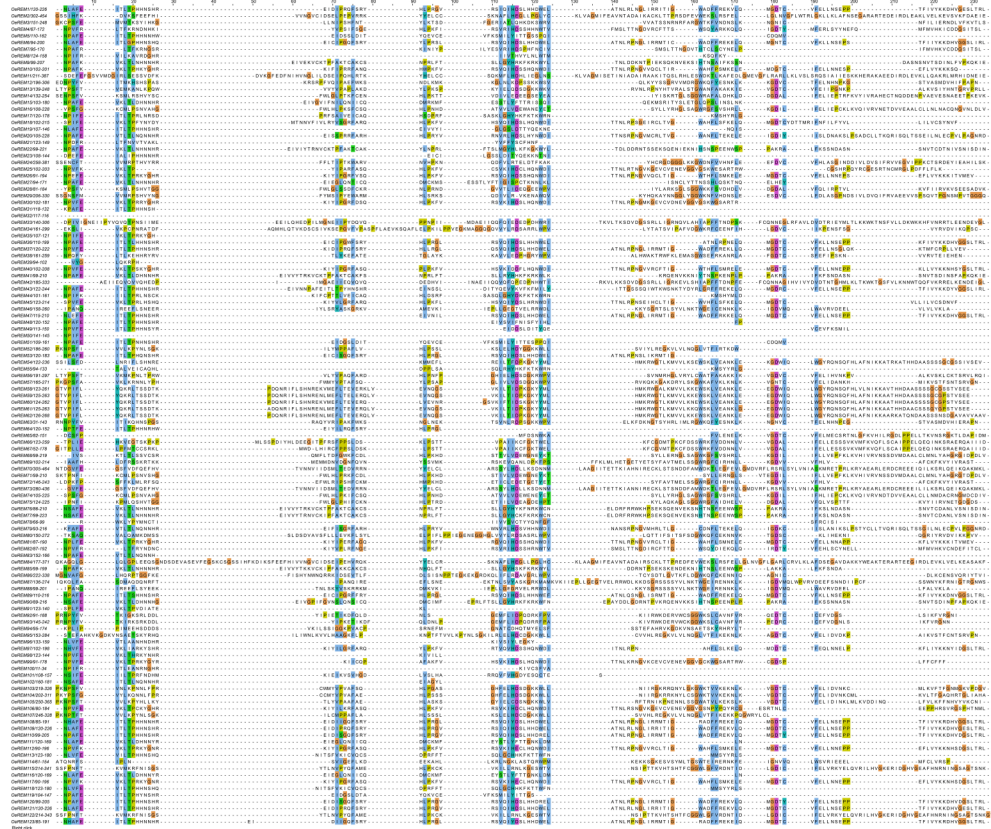

Supplement: Supplementary Materials — Table S1: the primers used in the qRT-PCR experiments in this study. Table S2: the overview of B3 superfamily in olive. Table S3: duplicated genes of B3 superfamily identified in olive. Table S4: the duplicated genes of B3 superfamily between olive and Arabidopsis and between olive and rice. Table S5: the largest syntenic blocks between olive and Arabidopsis and between olive and rice. Table S6: hormone-related cis-elements identified in the B3 superfamily in olive. Table S7: the expression levels of duplicated genes of B3 superfamily in olive. Table S8: the Ka/Ks ratios of duplicated genes of B3 superfamily in olive. Figure S1: the multiple alignment of the B3 proteins in the REM family of B3 genes in olive showing the two domains (a, b) located in the REM family. Figure S2: the multiple alignment of the B3 proteins in the RAV family of B3 genes in olive showing the AP2 and B3 domains. [file 6051511.f1.zip › Figure.S1.pdf]
