# Supplementary material for: Genome-Wide Analysis of the Molecular Functions of B3 Superfamily in Oil Biosynthesis in Olive (Olea europaea L.)
Source: Biomed Res Int. 2023 Feb 14;2023:6051511. doi: 10.1155/2023/6051511 (PMC9943606; doi:10.1155/2023/6051511)
Supplement: Supplementary Materials — Table S1: the primers used in the qRT-PCR experiments in this study. Table S2: the overview of B3 superfamily in olive. Table S3: duplicated genes of B3 superfamily identified in olive. Table S4: the duplicated genes of B3 superfamily between olive and Arabidopsis and between olive and rice. Table S5: the largest syntenic blocks between olive and Arabidopsis and between olive and rice. Table S6: hormone-related cis-elements identified in the B3 superfamily in olive. Table S7: the expression levels of duplicated genes of B3 superfamily in olive. Table S8: the Ka/Ks ratios of duplicated genes of B3 superfamily in olive. Figure S1: the multiple alignment of the B3 proteins in the REM family of B3 genes in olive showing the two domains (a, b) located in the REM family. Figure S2: the multiple alignment of the B3 proteins in the RAV family of B3 genes in olive showing the AP2 and B3 domains. [file Figure.S2.pdf › Figure.S2.pdf]

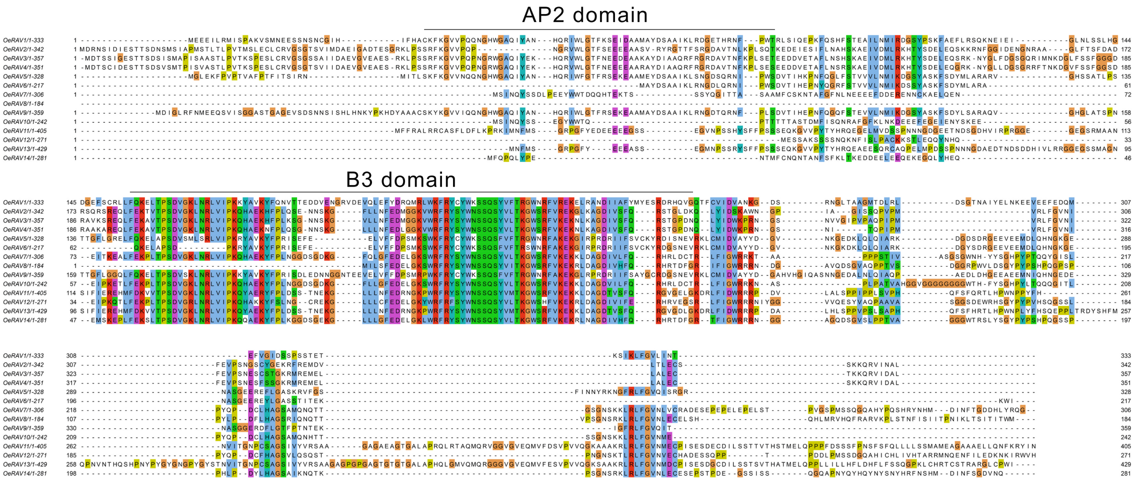
AP2 domain
B3 domain
OeRAV1/1-333
OeRAV2/1-342
OeRAV3/1-357
OeRAV4/1-351
OeRAV5/1-328
OeRAV6/1-217
OeRAV7/1-306
OeRAV8/1-184
OeRAV9/1-359
OeRAV10/1-242
OeRAV11/1-405
OeRAV12/1-271
OeRAV13/1-429
OeRAV14/1-281
